# COVID-19 Vaccine Booster Uptake and Effectiveness Among US Adults With Cancer

**DOI:** 10.1001/jamaoncol.2025.2020

**Published:** 2025-07-17

**Authors:** Jacek Skarbinski, Eric P. Elkin, Yonah C. Ziemba, Elham Kazemian, Brigid M. Wilson, Hinnah Siddiqui, Cheryl B. Schleicher, Crystal A. Hsiao, Joshua R. Nugent, Karen L. Reckamp, Akil Merchant, James M. Crawford, David A. Zidar, Lawrence H. Kushi, Jane C. Figueiredo

**Affiliations:** 1 Division of Research, Kaiser Permanente Northern California, Pleasanton; 2 Department of Infectious Diseases, Oakland Medical Center, Kaiser Permanente Northern California, Oakland; 3 Physician Researcher Program, Kaiser Permanente Northern California, Pleasanton; 4 The Permanente Medical Group, Kaiser Permanente Northern California, Pleasanton; 5 Northwell Health, Feinstein Institutes for Medical Research, Manhasset, New York; 6 Department of Medicine, Samuel Oschin Cancer Center, Cedars-Sinai Medical Center, Los Angeles, California; 7 Louis Stokes Cleveland Veterans Affairs Medical Center, Cleveland, Ohio; 8 Case Western Reserve University School of Medicine, Cleveland, Ohio

## Abstract

**Importance:**

Persons with cancer are at increased risk of severe COVID-19 infection, but the additional benefit of COVID-19 boosters is unclear.

**Objective:**

To assess COVID-19 vaccine effectiveness (VE) and number needed to vaccinate (NNV) among persons with cancer of an additional dose of the monovalent COVID-19 vaccine.

**Design, Setting, and Participants:**

Retrospective cohort study conducted in 4 health care systems in the US among persons with cancer receiving chemotherapy or immunotherapy. Statistical analysis was conducted between March 2023 and August 2024.

**Exposures:**

Receipt of an additional dose of the monovalent COVID-19 vaccine before January 1, 2022, with follow-up until August 31, 2022, and the bivalent COVID-19 vaccine from September 1, 2022, to August 31, 2023.

**Main Outcomes and Measures:**

COVID-19 hospitalization, diagnosed COVID-19, and COVID-19–related intensive care unit (ICU) admission.

**Results:**

Among 72 831 persons with cancer (17 922 female individuals [24.6%]), 69% received a monovalent booster by January 1, 2022. During 34 006 person-years of follow-up, the COVID-19 hospitalization rate was 30.5 per 1000 person-years among patients who received a monovalent booster vs 41.9 per 1000 person-years among patients who received the primary series alone, with an adjusted VE of 29.2% (95% CI, 19.9%-37.3%) and NNV to prevent 1 COVID-19 hospitalization of 166 (95% CI, 130-244). There was also significant VE to prevent diagnosed COVID-19 (8.5% [95% CI, 3.7%-13.0%]) and COVID-19–related ICU admission (35.6% [95% CI, 20.0%-48.3%]). Among 88 417 persons with cancer (24 589 female individuals [27.8%]) with 81 027 person-years of follow-up during the bivalent period, patients who received this booster (38%) had a COVID-19 hospitalization rate of 13.4 per 1000 person-years vs 21.7 per 1000 person-years among persons who did not receive a bivalent vaccine, with an adjusted VE of 29.9% (95% CI, 19.4%-39.1%) and NNV to prevent 1 COVID-19 hospitalization of 310 (95% CI, 237-479); the adjusted VE was 30.1% (95% CI, 7.7%-47.0%) to prevent COVID-19–related ICU admission.

**Conclusions and Relevance:**

In this retrospective cohort study, COVID-19 booster vaccinations were associated with significant protection against severe COVID-19, with a favorable NNV among persons with cancer. However, uptake of COVID-19 vaccine boosters was low, and interventions are therefore justified to increase COVID-19 uptake in this high-risk population.

## Key Points

**Question:**

How effective were additional doses of COVID-19 vaccines at preventing COVID-19 hospitalizations among persons with cancer from January 2022 to August 2023?

**Findings:**

In a retrospective cohort study among persons with cancer in the US, additional monovalent COVID-19 vaccines reduced COVID-19 hospitalizations by 29%, with a number needed to vaccinate of 166 in January to August 2022, and a bivalent COVID-19 vaccine reduced COVID-19 hospitalizations by 30%, with a number needed to vaccinate of 310 in September 2022 to August 2023.

**Meaning:**

In both time periods, COVID-19 vaccination was associated with protection from severe COVID-19 among persons with cancer.

## Teaser

This retrospective cohort study examines the uptake and effectiveness of COVID-19 booster vaccines in preventing hospitalizations due to severe COVID-19 infection among patients with cancer.

## Introduction

Persons with cancer, especially patients receiving anticancer treatments, are at increased risk of severe illness and mortality from SARS-CoV-2 infection.^1^ In November 2021, the US Centers for Disease Control and Prevention recommended additional COVID-19 vaccine doses for immunocompromised individuals, including patients with cancer who had completed their primary vaccine series. Since then, annual vaccination with an additional dose after 6 months has been recommended for persons who are moderately or severely immunocompromised.^2^

Because phase 3 COVID-19 vaccine trials excluded patients with cancer,^3,4^ the initial evidence to support the administration of additional vaccine boosters for patients with cancer was derived predominantly from immunologic studies showing reduced immune responses in patients with cancer^5,6,7,8,9,10,11^ and observational studies of vaccine effectiveness (VE).^12,13,14,15,16,17^ These data showed suboptimal immune responses among patients with cancer who received the primary 2-dose vaccination schedule and lower VE and increased risk of COVID-19–related intensive care unit (ICU) admission and mortality.

With the transition of COVID-19 into an endemic disease, it is crucial to recognize that patients with cancer will be at a persistently heightened risk of severe illness, and therefore ongoing assessment of vaccination usage and outcomes remains vital for informing effective prevention efforts.^18^ VE studies should take into account the diverse characteristics of patients and their disease conditions, including primary tumor site, stage of disease, treatment regimen, underlying medical conditions, and demographic factors, such as age and sex.^19^ This real-world data study uses electronic health record data obtained from 4 National Cancer Institute–funded Serological Sciences Network sites in the US to assess VE of an additional dose of COVID-19 vaccine for preventing COVID-19 hospitalization, diagnosed COVID-19, and COVID-19–related ICU admission among persons with cancer during 2 time periods: (1) the monovalent booster period (an additional dose of the monovalent COVID-19 vaccine before January 1, 2022, with follow-up until August 31, 2022) and (2) the bivalent vaccine period (bivalent COVID-19 vaccine from September 1, 2022-August 31, 2023).^20^

## Methods

### Study Design

We conducted a retrospective cohort study using data from 4 health care systems (Cedars-Sinai Health System, Kaiser Permanente Northern California [KPNC], Northwell Health, and Veterans Health Administration), with varied catchment areas, sizes, and patient characteristics (eTables 1-3 in Supplement 1). Data were analyzed independently within each site and the summary results from each site were combined in a meta-analysis to produce summary effect estimates. Individual patient-level data were not combined across sites. Each site had approval from their local institutional review board for this research with a waiver of informed consent.

### Data Harmonization, Variable Definitions, and Study Population

We developed a harmonized plan to ensure consistency of data extraction, definitions, and analysis at all sites. Each site extracted data to construct a cohort for each time period using a common data dictionary with *International Classification of Diseases, Ninth Revision *(*ICD-9*) and* International Statistical Classification of Diseases and Related Health Problems, Tenth Revision* (*ICD-10*) diagnosis codes and cancer classifications adapted from studies of similar populations^16,17^ and a standardized list of medications (eTables 4 and 5 in Supplement 1).

In each health care system, we identified adults aged 18 to 89 years and alive as of the index date, defined as January 1, 2022, for the monovalent vaccine period or September 1, 2022, for the bivalent vaccine period. At Cedars-Sinai, Northwell, and Veterans Health Administration, all patients had at least 1 inpatient, outpatient, virtual care, or emergency department encounter in the 12 months prior to the index date and at least 1 encounter in the 12 months after the index date. In KPNC, a closed, integrated health care system, inclusion criteria required membership for 12 months prior to the index date and at least 1 month of membership between the index date and end of observation.

Persons with cancer were defined as patients who had a cancer diagnosis code and had received either cytotoxic chemotherapy or immunotherapy (using Cancer Medications Enquiry Database [CanMED] classification) in the year before the cohort start date. Persons with skin cancer alone (including melanoma) and patients undergoing only surgical procedures or radiation were excluded. We only included systemic corticosteroid use if it was used for cancer treatment. All sites obtained vaccination records for receipt of all doses of COVID-19 monovalent vaccines (BNT162b2 [Pfizer/BioNTech], mRNA-1273 [Moderna/National Institutes of Health], or Ad26.COV2.s [Janssen]) before January 1, 2022, and COVID-19 bivalent vaccines (Pfizer or Moderna) from September 1, 2022, to August 31, 2023.

For the monovalent booster period, the primary exposure of interest was receipt of 1 COVID-19 monovalent booster dose (in addition to completion of the primary series) compared with patients who had completed the primary series only as of January 1, 2022. Completed primary series included persons who received 1 dose of Ad26.COV2.s or 2 doses of BNT162b2 at least 14 days apart or mRNA-1273 at least 21 days apart. Completed primary series plus additional dose included persons who completed a primary series and received at least 1 additional dose of any COVID-19 vaccine (Ad26.COV2.s, BNT162b2, or mRNA-1273) at least 28 days after completing the primary series. For the bivalent vaccine period, the primary exposure of interest was receipt of 1 bivalent COVID-19 vaccine dose (Pfizer or Moderna) compared with patients who did not receive a bivalent booster dose from September 1, 2022, to August 31, 2023. Persons who received the Novavax COVID-19 vaccine or COVID-19 vaccines not approved in the US were excluded.

The primary outcome was COVID-19 hospitalization, defined as an inpatient admission with an associated COVID-19 diagnosis or related code (eg, pneumonia, sepsis) (eTable 6 in Supplement 1) and positive SARS-CoV-2 nucleic acid amplification test results at any time 14 days before to 7 days after hospital admission. Secondary outcomes were diagnosed COVID-19 and COVID-19 hospitalization requiring ICU admission at any time during the hospitalization (COVID-19–related ICU admission). Diagnosed COVID-19 was defined as the first positive result on a SARS-CoV-2 nucleic acid amplification test or a COVID-19 diagnosis code from a care encounter during the study period.

To provide context for the results of patients with cancer, we also included data on the nonimmunocompromised population as a reference. These were patients who did not have any diagnoses of cancer, autoimmune diseases, solid organ transplant, or other immunodeficiency and had not received any cytotoxic chemotherapy, immunotherapy, or systemic corticosteroids in the past year. We present results for the entire nonimmunocompromised population as well as stratified by age (<65 years, ≥65 years).

### Statistical Analysis

Statistical analysis was conducted between March 2023 and August 2024. All analyses were stratified by cancer and medication type (cytotoxic chemotherapy, immunotherapy, systemic corticosteroids). Patients may have been included in more than 1 cancer or medication stratum. The nonimmunocompromised group was analyzed in parallel to provide a reference for VE of COVID-19 vaccination in the nonimmunocompromised population.

Each site performed Cox proportional hazards modeling of their data, using R statistical software (R Foundation). Three proportional hazards models (1 per outcome) were performed for each stratum of cancer type, medication type, and/or nonimmunocompromised group. Time to first outcome during the follow-up period was modeled while accounting for censoring events: end of the observation period, death, receipt of another COVID-19 vaccination, or loss of health plan membership at KPNC only.

For the monovalent booster period cohort, models included vaccination status as of January 1, 2022 (ie, completed primary series or primary series plus 1 additional monovalent COVID-19 vaccination), as well as age group in years (18-49, 50-64, 65-74, 75-89), sex (male, female), race and ethnicity (Hispanic of any race, Asian or Pacific Islander, Black, White, other [American Indian, multiracial, other race (not specified), unknown race]), and Charlson Comorbidity Index score (≥3, <3; scores range from 0-29, with higher scores indicating greater comorbidity)^21,22^ based on diagnosis codes recorded in the year prior. Demographic data were collected from the electronic health record. For the bivalent vaccine period cohort, models included a time-dependent variable for receipt of bivalent COVID-19 vaccine and controlled for the same variables as the monovalent booster period cohort plus vaccination status on September 1, 2022. The proportional hazards assumption was tested using Schoenfeld residuals and Kaplan-Meier curves for each model at each site. R programs were created using a federated approach to ensure uniformity of data definitions and output.

Meta-analyses based on the adjusted β coefficients and their standard errors for the comparisons of interest from the proportional hazards models from each health care system were analyzed centrally at KPNC. The summary β coefficient was calculated using the inverse variance–weighted mean of the individual β coefficients, and the summary standard error of the β was calculated from the inverse square root of the sum of the weights.^23^ These methods assume homogeneity of results across the 4 health care systems because of the small number of sites.^24^ VE of additional vaccinations was calculated as 1 − hazard ratio and reported as a percentage. Number needed to vaccinate (NNV) to prevent 1 outcome was calculated as 1 ÷ absolute risk reduction.^25,26^

## Results

### Effectiveness of Monovalent COVID-19 Vaccine Booster

Of 72 831 persons with cancer (17 922 female individuals [24.6%]) with 34 006 person-years of follow-up and 4 870 138 nonimmunocompromised persons with 2 511 721 person-years of follow-up during the monovalent COVID-19 vaccine booster period, 69% of persons with cancer and 49% of nonimmunocompromised persons received a monovalent booster before January 1, 2022 (Table 1). Among persons with cancer, the COVID-19 hospitalization rate was 30.5 per 1000 person-years for patients who received a monovalent booster vs 41.9 per 1000 person-years for patients who completed a primary series only (Table 2, Figure). Among persons with cancer, the VE of a monovalent booster to prevent COVID-19 hospitalization was 29.2% (95% CI, 19.9%-37.3%) with an NNV to prevent 1 COVID-19 hospitalization of 166 (95% CI, 130-244). VE and NNV were similar among subgroups of persons with solid malignant neoplasms (VE, 24.3% [95% CI, 13.2%-33.9%]; NNV, 215 [95% CI, 154-395]) and hematologic malignant neoplasms (VE, 29.8% [95% CI, 15.0%-42.1%]; NNV, 115 [81-230]). Further subgroup analyses showed significant VE among persons with cancer of the lip, oral cavity, or pharynx; other gastrointestinal organs; lung; other respiratory and intrathoracic organs; bone; mesothelial or other soft tissue; prostate; and myeloma. Persons receiving cytotoxic chemotherapy, immunotherapy, or systemic corticosteroids for cancer treatment had significantly reduced rates of COVID-19 hospitalization with monovalent vaccination. In comparison, the COVID-19 hospitalization rate was 2.1 per 1000 person-years among nonimmunocompromised persons who received a monovalent booster vs 3.6 per 1000 person-years among patients who completed a primary series only, with an overall VE of 50.8% (95% CI, 48.3%-53.2%) but an NNV of 1107 (95% CI, 1058-1164).

**Table 1.** Characteristics of Monovalent COVID-19 Vaccine Booster Period (January 1, 2022-August 31, 2022) Cohort Combined From 4 Health Care Systems

Characteristic	All cancer^a^			Solid malignant neoplasms^a^			Hematologic malignant neoplasms^a^			Nonimmunocompromised^a^		
	Primary series, No. (%)^b^		Percentage with 1 additional dose	Primary series, No. (%)		Percentage with 1 additional dose	Primary series, No. (%)		Percentage with 1 additional dose	Primary series, No. (%)		Percentage with 1 additional dose
	Primary series only	One additional dose		Primary series only	One additional dose		Primary series only	One additional dose		Primary series only	One additional dose	
Total No.	22 566	50 265	69	19 131	42 910	69	6403	15 046	70	2 483 954	2 386 184	49
Age, y												
18-49	2237 (10)	2539 (5)	53	1750 (9)	2012 (5)	53	665 (10)	673 (4)	50	1 172 300 (47)	703 233 (29)	37
50-64	5656 (25)	9624 (19)	63	4776 (25)	8039 (19)	63	1491 (23)	2658 (18)	64	649 840 (26)	642 713 (27)	50
65-74	8269 (37)	20 804 (41)	72	7160 (37)	18 009 (42)	72	2254 (35)	6077 (40)	73	397 033 (16)	617 417 (26)	61
75-89	6404 (28)	17 298 (34)	73	5445 (28)	14 850 (35)	73	1993 (31)	5638 (37)	74	264 781 (11)	422 821 (18)	61
Female												
Yes	5680 (25)	12 242 (24)	68	4823 (25)	10 399 (24)	68	1208 (19)	2650 (18)	69	787 189 (32)	836 209 (35)	52
No	16 886 (75)	38 023 (76)	69	14 308 (75)	32 511 (76)	69	5195 (81)	12 396 (82)	70	1 696 765 (68)	1 549 975 (65)	48
Race and ethnicity												
Hispanic	2093 (9)	4028 (8)	66	1717 (9)	3350 (8)	66	577 (9)	1124 (7)	66	406 313 (16)	271 736 (11)	40
Asian or Pacific Islander	1291 (6)	3373 (7)	72	1047 (5)	2764 (6)	73	312 (5)	831 (6)	73	257 452 (10)	383 324 (16)	60
Black	3607 (16)	7578 (15)	68	3103 (16)	6666 (16)	68	1082 (17)	2354 (16)	69	331 942 (13)	260 452 (11)	44
White	14 272 (63)	33 007 (66)	70	12 166 (64)	28 164 (66)	70	4050 (63)	10 059 (67)	71	1 256 888 (51)	1 298 106 (54)	51
Other or unknown^c^	1303 (6)	2279 (5)	64	1098 (6)	1966 (5)	64	382 (6)	678 (5)	64	231 359 (9)	172 566 (7)	43
Charlson Comorbidity Index score ≥3^d^												
Yes	16 547 (73)	39 080 (78)	70	14 366 (75)	34 133 (80)	70	4444 (69)	11 188 (74)	72	182 621 (7)	266 810 (11)	59
No	6019 (27)	11 185 (22)	65	4765 (25)	8777 (20)	65	1959 (31)	3858 (26)	66	2 301 333 (93)	2 119 374 (89)	48

^a^ See eTables 4 and 5 in Supplement 1 for definitions.

^b^ As of index date January 1, 2022. Primary series was 1 dose of Ad26.COV2.s (Janssen) or 2 doses of BNT162b2 (Pfizer/BioNTech; at least 14 days apart) or mRNA-1273 (Moderna/National Institutes of Health; at least 21 days apart). Additional vaccination must have been at least 28 days after prior vaccination.

^c^ Included American Indian, multiracial, and other race (not specified).

^d^ Scores range from 0-29, with higher scores indicating greater comorbidity.

**Table 2.** Effectiveness of 1 Additional Dose of Monovalent COVID-19 Vaccination (Booster) Compared With Primary Series Only to Prevent COVID-19 Hospitalization in Persons With Cancer and Nonimmunocompromised Persons, January 1, 2022-August 31, 2022^a^

Cancer type^b^	No. of sites contributing to pooled analysis	Events, No./total No. of people (rate per 1000 person-years)^c^		Pooled adjusted HR^d^ (95% CI)	Effectiveness of 1 additional vaccination, % (95% CI)	No. needed to vaccinate (95% CI)
		Primary series only	One additional vaccination			
All cancer (solid or hematologic)	4	433/22 566 (41.9)	716/50 265 (30.5)	0.71 (0.63 to 0.80)	29.2 (19.9 to 37.3)	166 (130 to 244)
Solid malignant neoplasms	4	341/19 131 (38.9)	613/42 910 (30.5)	0.76 (0.66 to 0.87)	24.3 (13.2 to 33.9)	215 (154 to 395)
Lip/oral cavity/pharynx	2	22/893 (53.9)	27/2112 (27.2)	0.49 (0.27 to 0.86)	51.4 (14.0 to 72.5)	73 (52 to 271)
Colorectal	4	42/2215 (41.7)	70/4208 (34.9)	0.79 (0.53 to 1.19)	20.6 (−18.8 to 46.9)	236 (103 to NB)
Other gastrointestinal^e^	4	55/2359 (54.4)	92/5694 (36.3)	0.62 (0.44 to 0.88)	37.5 (12.0 to 55.6)	100 (67 to 313)
Lung	4	72/2742 (61.9)	110/6122 (39.1)	0.64 (0.47 to 0.87)	35.8 (12.8 to 52.8)	92 (62 to 260)
Other respiratory and intrathoracic organs	2	14/541 (60.3)	19/1188 (34.0)	0.47 (0.23 to 0.96)	52.9 (4.3 to 76.9)	64 (43 to 802)
Bone/mesothelial/soft tissue	3	26/943 (60.5)	28/2215 (27.6)	0.41 (0.24 to 0.71)	58.9 (28.9 to 76.3)	57 (44 to 117)
Breast	4	29/2199 (29.4)	39/5034 (16.9)	0.61 (0.37 to 1.02)	38.8 (−2.0 to 63.3)	177 (108 to NB)
Gynecological	4	13/856 (33.4)	21/1662 (28.2)	1.06 (0.50 to 2.26)	−6.0 (−126.3 to 50.4)	NB (120 to NB)
Prostate	4	63/3916 (34.0)	121/10 072 (25.4)	0.70 (0.52 to 0.96)	29.5 (4.1 to 48.3)	201 (123 to 1468)
Urinary tract	3	44/2454 (39.7)	115/6104 (39.9)	0.97 (0.68 to 1.39)	2.9 (−38.9 to 32.1)	1784 (159 to NB)
Central nervous system	4	16/690 (53.7)	22/1293 (36.9)	0.65 (0.33 to 1.27)	34.9 (−27.1 to 66.6)	109 (56 to NB)
Endocrine glands	3	20/1036 (42.2)	29/2210 (27.8)	0.59 (0.33 to 1.07)	41.1 (−6.7 to 67.5)	117 (71 to NB)
Hematologic malignant neoplasms	4	174/6403 (59.5)	300/15 046 (43.8)	0.70 (0.58 to 0.85)	29.8 (15.0 to 42.1)	115 (81 to 230)
Leukemia	4	67/2838 (51.0)	121/6410 (40.7)	0.75 (0.55 to 1.01)	25.3 (−1.2 to 44.9)	158 (89 to NB)
Lymphoma	4	69/2267 (68.0)	135/5136 (58.8)	0.84 (0.62 to 1.14)	16.0 (−14.1 to 38.2)	189 (79 to NB)
Myeloma	4	45/1391 (72.2)	71/3656 (44.4)	0.58 (0.39 to 0.86)	42.3 (14.5 to 61.1)	67 (46 to 198)
Myelodysplastic syndromes	4	27/692 (89.8)	39/1563 (55.2)	0.64 (0.38 to 1.06)	36.0 (−6.3 to 61.5)	64 (37 to NB)
All cancer medications						
Cytotoxic chemotherapy	4	183/8205 (52.1)	248/15 662 (35.0)	0.69 (0.56 to 0.83)	31.4 (16.6 to 43.5)	125 (90 to 236)
Immunotherapy	4	339/17 310 (42.4)	602/40 532 (31.9)	0.73 (0.64 to 0.84)	27.1 (16.4 to 36.4)	177 (131 to 293)
Systemic corticosteroids	4	277/11 111 (57.4)	462/23 814 (43.2)	0.74 (0.63 to 0.86)	26.4 (14.2 to 36.8)	135 (96 to 251)
Solid malignant neoplasm medications						
Cytotoxic chemotherapy	4	149/7269 (47.6)	210/13 998 (32.9)	0.68 (0.55 to 0.85)	31.6 (15.2 to 44.8)	135 (95 to 282)
Immunotherapy	4	261/14 312 (39.5)	506/33 979 (31.9)	0.78 (0.67 to 0.91)	22.2 (9.3 to 33.2)	232 (155 to 556)
Systemic corticosteroids	4	227/9675 (53.8)	395/20 837 (42.0)	0.76 (0.64 to 0.89)	24.5 (10.7 to 36.1)	155 (105 to 357)
Hematologic malignant neoplasm medications and transplants						
Cytotoxic chemotherapy	4	65/1866 (83.7)	98/3661 (61.5)	0.74 (0.54 to 1.03)	25.6 (−2.7 to 46.2)	96 (53 to NB)
Immunotherapy	4	154/5581 (60.2)	283/13 333 (46.7)	0.74 (0.61 to 0.91)	25.9 (9.2 to 39.5)	131 (86 to 369)
Systemic corticosteroids	4	114/2986 (90.4)	201/6818 (68.0)	0.70 (0.56 to 0.89)	29.7 (10.9 to 44.5)	77 (51 to 211)
Hematopoietic stem cell transplant	4	14/702 (47.8)	22/1668 (31.2)	0.68 (0.33 to 1.38)	32.3 (−38.2 to 66.9)	132 (63 to NB)
Nonimmunocompromised persons						
All	4	4229/2 483 954 (3.6)	2804/2 386 184 (2.1)	0.49 (0.47 to 0.52)	50.8 (48.3 to 53.2)	1107 (1058 to 1164)
Age, y						
<65	4	1881/1 822 140 (2.2)	845/1 345 946 (1.1)	0.51 (0.47 to 0.55)	49.1 (44.7 to 53.2)	1822 (1682 to 2003)
≥65	4	2348/661 814 (6.8)	1959/1 040 238 (3.7)	0.49 (0.46 to 0.52)	51.0 (47.9 to 53.9)	580 (549 to 617)

Abbreviations: HR, hazard ratio; NB, no benefit.

^a^ Pooled results from 4 health care systems.

^b^ See eTables 4 and 5 in Supplement 1 for definitions. Patients may have had more than 1 diagnosis and/or received more than 1 type of medication and were included in the analysis of each stratum for which they qualified.

^c^ As of index date January 1, 2022. Primary series was 1 dose of Ad26.COV2.s (Janssen) or 2 doses of BNT162b2 (Pfizer/BioNTech; at least 14 days apart) or mRNA-1273 (Moderna/National Institutes of Health; at least 21 days apart). Additional vaccination must have been at least 28 days after prior vaccination.

^d^ Each site ran a Cox proportional hazards model on their own data, including vaccination status as of the index date (January 1, 2022), adjusting for age, sex, race and ethnicity, and Charlson Comorbidity Index score. Pooled HRs were calculated from the inverse variance–weighted fixed effects of the proportional hazards model results from each site.^18^

^e^ Other gastrointestinal cancers included esophageal, pancreatic, gall bladder, anus, anal canal, and anorectum.

**Figure.**
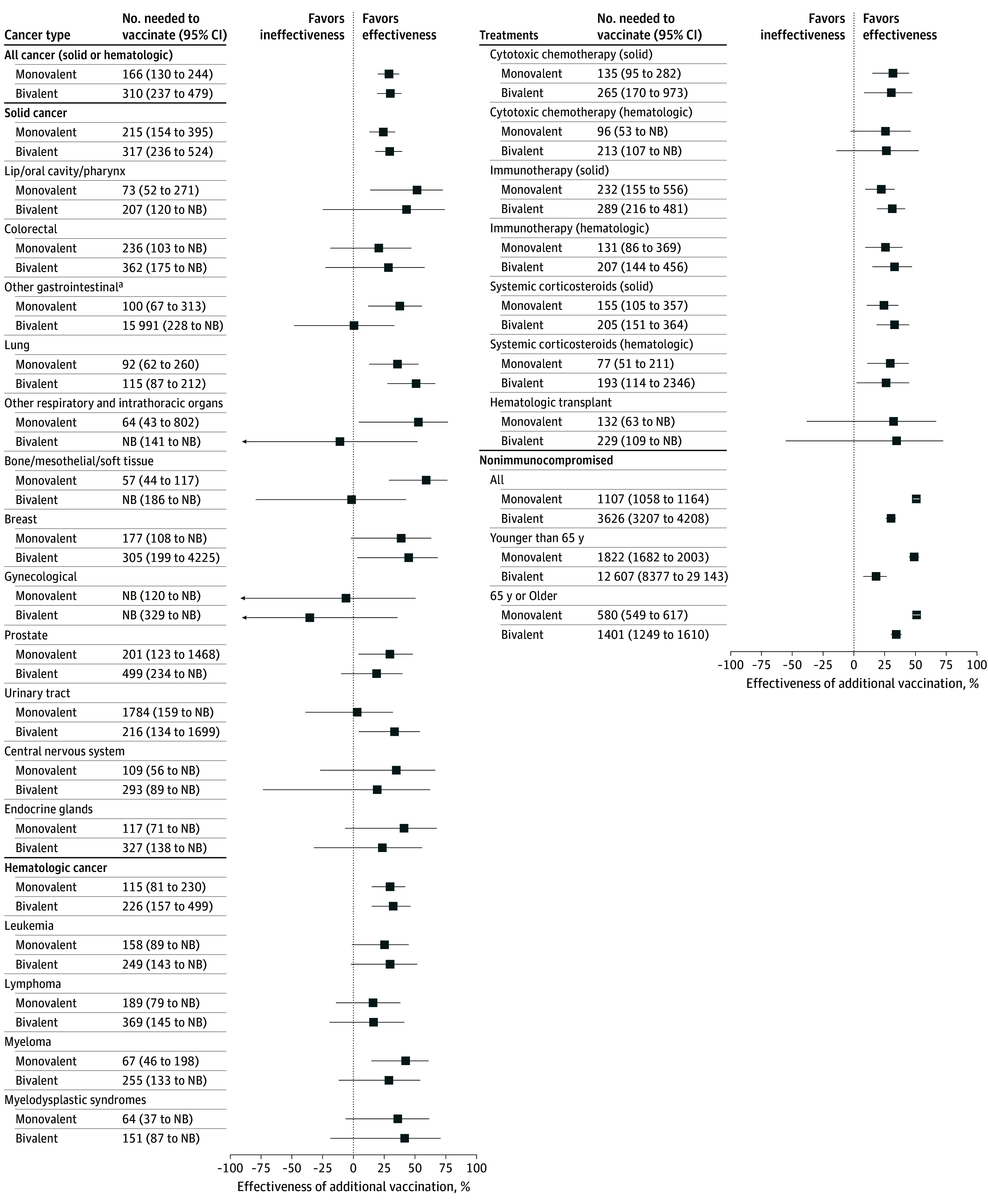
Pooled Vaccine Effectiveness (VE) and Number Needed to Vaccinate to Prevent COVID-19 Hospitalization in Persons With Cancer and Nonimmunocompromised Persons Who Received 1 Additional Dose of COVID-19 Vaccine VE = (1 – pooled HR); HRs calculated from inverse variance–weighted fixed effects of multivariate Cox proportional hazards model (controlled for age, sex, race and ethnicity, Charlson Comorbidity Index score) from each site. For bivalent analysis, patients' vaccination status included as of index date. Patients may have had ≥1 diagnosis and/or received ≥1 treatment in past year and included in analysis of each qualifying stratum. HR indicates hazard ratio; and NB, no benefit. ^a^Esophageal, pancreatic, gall bladder, anus, anal canal, and anorectum.

Among persons with cancer, the rate of diagnosed COVID-19 was 216.3 per 1000 person-years for patients who received a monovalent booster and 241.8 per 1000 person-years for patients who completed a primary series only, with a VE of 8.5% (95% CI, 3.7%-13.0%) and an NNV of 110 (95% CI, 71-253) to prevent 1 episode of diagnosed COVID-19 (eTable 7 and eFigure 1 in Supplement 1). VE and NNV were similar among persons with solid malignant neoplasms (VE, 6.1% [95% CI, 0.8%-11.2%]; NNV, 157 [95% CI, 85-1254]) and hematologic malignant neoplasms (VE, 10.2% [95% CI, 2.2%-17.7%]; NNV, 76 [95% CI, 44-367]). The VE of a monovalent booster to prevent diagnosed COVID-19 among nonimmunocompromised persons was 5.3% (95% CI, 4.6%-6.0%) with an NNV of 302 (95% CI, 267-348).

The rate of COVID-19–related ICU admission among persons with cancer was 9.2 per 1000 person-years among patients who received a monovalent booster and 13.3 per 1000 person-years for patients who completed a primary series only, with a VE of 35.6% (95% CI, 20.0%-48.3%) and an NNV of 423 (95% CI, 312-756) to prevent 1 COVID-19–related ICU admission (eTable 8 and eFigure 2 in Supplement 1). The NNV to prevent 1 COVID-19–related ICU admission was 578 (95% CI, 372-1939) among persons with solid malignant neoplasms and 252 (95% CI, 176-616) among persons with hematologic malignant neoplasms. Although the VE among nonimmunocompromised persons to prevent COVID-19–related ICU admission was 53.0% (95% CI, 47.8%-57.7%), the NNV was 4621 (95% CI, 4245-5126), as the rate of COVID-19–related ICU admission among immunocompromised persons was low (0.4/1000 person-years among patients who received a monovalent booster vs 0.8/1000 person-years among patients who completed a primary series only).

### Effectiveness of Bivalent COVID-19 Vaccine

We included 88 417 persons with cancer (24 589 female individuals) with 81 027 person-years of follow-up and 6 408 045 nonimmunocompromised persons with 6 191 976 person-years of follow-up during the bivalent COVID-19 vaccine period. Bivalent vaccine uptake was higher among persons with cancer (38%) than nonimmunocompromised persons (24%) (Table 3). Among persons with cancer, the COVID-19 hospitalization rate was lower among patients who received a bivalent vaccine (13.4/1000 person-years) vs patients who did not receive a bivalent vaccine (21.7/1000 person-years) with a VE of 29.9% (95% CI, 19.4%-39.1%) and an NNV to prevent 1 COVID-19 hospitalization of 310 (95% CI, 237-479) (Table 4). Findings were similar among persons with solid malignant neoplasms (VE, 29.5% [95% CI, 17.9%-39.5%]; NNV, 317 [95% CI, 236-524]) and hematologic malignant neoplasms (VE, 32.3% [95% CI, 14.7%-46.3%]; NNV, 226 [95% CI, 157-499]). The VE was significant for preventing COVID-19 hospitalization among persons with cancer of the lung, breast, and urinary tract and among persons receiving cytotoxic chemotherapy, immunotherapy, or systemic corticosteroids. Among nonimmunocompromised persons, the COVID-19 hospitalization rate was lower among patients who received a bivalent vaccine (1.6/1000 person-years) than patients who did not receive a bivalent vaccine (1.8/1000 person-years), with a VE of 30.1% (95% CI, 25.9%-34.0%) but an NNV of 3626 (95% CI, 3207-4208).

**Table 3.** Characteristics of Bivalent COVID-19 Vaccine Period (September 1, 2022-August 31, 2023) Cohort Combined From 4 Health Care Systems

Characteristic	All cancer^a^			Solid malignant neoplasms^a^			Hematologic malignant neoplasms^a^			Nonimmunocompromised^a^		
	Bivalent vaccine, No. (%)		Percentage with bivalent vaccine	Bivalent vaccine, No. (%)		Percentage with bivalent vaccine	Bivalent vaccine, No. (%)		Percentage with bivalent vaccine	Bivalent vaccine, No. (%)		Percentage with bivalent vaccine
	No	Received		No	Received		No	Received		No	Received	
Total No.	54 711	33 706	38	46 544	28 645	38	15 237	9920	39	4 838 493	1 569 552	24
Age, y												
18-49	5398 (10)	1447 (4)	21	4219 (9)	1147 (4)	21	1533 (10)	392 (4)	20	2 223 616 (46)	429 666 (27)	16
50-64	12 936 (24)	6001 (18)	32	10 928 (23)	4926 (17)	31	3251 (21)	1694 (17)	34	1 258 958 (26)	414 465 (26)	25
65-74	18 770 (34)	13 143 (39)	41	16 267 (35)	11 249 (39)	41	5022 (33)	3757 (38)	43	766 466 (16)	409 937 (26)	35
75-89	17 607 (32)	13 115 (39)	43	15 130 (33)	11 323 (40)	43	5431 (36)	4077 (41)	43	589 453 (12)	315 484 (20)	35
Female												
Yes	15 244 (28)	9345 (28)	38	13 035 (28)	7888 (28)	38	3130 (21)	2135 (22)	41	1 653 878 (34)	572 941 (37)	26
No	39 467 (72)	24 361 (72)	38	33 509 (72)	20 757 (72)	38	12 107 (79)	7785 (78)	39	3 184 615 (66)	996 611 (63)	24
Race and ethnicity												
Hispanic	5043 (9)	2642 (8)	34	4171 (9)	2193 (8)	34	1336 (9)	765 (8)	36	738 079 (15)	170 275 (11)	19
Asian or Pacific Islander	3073 (6)	2604 (8)	46	2558 (5)	2128 (7)	45	694 (5)	649 (7)	48	459 488 (9)	264 656 (17)	37
Black	8044 (15)	5149 (15)	39	6902 (15)	4487 (16)	39	2354 (15)	1615 (16)	41	603 354 (12)	170 477 (11)	22
White	35 185 (64)	21 763 (65)	38	30 070 (65)	18 493 (65)	38	9897 (65)	6424 (65)	39	2 550 168 (53)	858 300 (55)	25
Other or unknown^b^	3366 (6)	1548 (5)	32	2843 (6)	1344 (5)	32	956 (6)	467 (5)	33	487 404 (10)	105 844 (7)	18
Charlson Comorbidity Index score ≥3^c^												
Yes	39 199 (72)	25 723 (76)	40	34 338 (74)	22 239 (78)	39	10 307 (68)	7405 (75)	42	333 227 (7)	186 721 (12)	36
No	15 512 (28)	7983 (24)	34	12 206 (26)	6406 (22)	34	4930 (32)	2515 (25)	34	4 505 266 (93)	1 382 831 (88)	23
Vaccination status^d^												
None/incomplete	11 861 (22)	245 (1)	2	10 040 (22)	207 (1)	2	3160 (21)	64 (1)	2	1 509 962 (31)	25 602 (2)	2
Primary series only	13 001 (24)	1099 (3)	8	10 943 (24)	922 (3)	8	3633 (24)	326 (3)	8	1 461 926 (30)	105 972 (7)	7
Primary series and 1 additional dose	20 794 (38)	10 207 (30)	33	17 803 (38)	8784 (31)	33	5768 (38)	2968 (30)	34	1 579 784 (33)	755 403 (48)	32
Primary series and 2 or 3 additional doses	9055 (17)	22 155 (66)	71	7758 (17)	18 732 (65)	71	2676 (18)	6562 (66)	71	286 821 (6)	682 575 (43)	70

^a^ See eTables 4 and 5 in Supplement 1 for definitions.

^b^ Included American Indian, multiracial, and other race (not specified).

^c^ Scores range from 0-29, with higher scores indicating greater comorbidity.

^d^ As of index date September 1, 2022. Incomplete status meant 1 dose of BNT162b2 (Pfizer/BioNTech) or mRNA-1273 (Moderna/National Institutes of Health) only. Primary series was 1 dose of Ad26.COV2.s (Janssen) or 2 doses of BNT162b2 (Pfizer/BioNTech; at least 14 days apart) or mRNA-1273 (Moderna/National Institutes of Health; at least 21 days apart). Additional vaccinations must have been at least 28 days after prior vaccination.

**Table 4.** Effectiveness of Bivalent COVID-19 Vaccine Compared With No Bivalent Vaccine to Prevent COVID-19 Hospitalization in Persons With Cancer and Nonimmunocompromised Persons, September 1, 2022-August 31, 2023^a^

Cancer type^b^	No. of sites contributing to pooled analysis	No. of events/total No. of people (rate per 1000 person-years)		Pooled adjusted HR^c^ (95% CI)	Effectiveness of bivalent vaccination, % (95% CI)	No. needed to vaccinate (95% CI)
		No bivalent vaccination	Bivalent vaccination			
All cancer (solid or hematologic)	4	1199/54 711 (21.7)	338/33 706 (13.4)	0.70 (0.61 to 0.81)	29.9 (19.4 to 39.1)	310 (237 to 479)
Solid malignant neoplasms	4	1007/46 544 (21.6)	283/28 645 (13.2)	0.71 (0.61 to 0.82)	29.5 (17.9 to 39.5)	317 (236 to 524)
Lip/oral cavity/pharynx	3	51/2256 (22.5)	9/1487 (8.2)	0.57 (0.26 to 1.25)	43.1 (−24.9 to 74.1)	207 (120 to NB)
Colorectal	4	103/5352 (19.8)	21/2687 (10.5)	0.72 (0.42 to 1.23)	28.1 (−22.6 to 57.8)	362 (175 to NB)
Other gastrointestinal^d^	3	137/5575 (26.7)	50/3477 (19.8)	1.00 (0.67 to 1.48)	0.5 (−48.1 to 33.1)	15 991 (228 to NB)
Lung	4	206/6562 (34.6)	37/3771 (13.5)	0.49 (0.34 to 0.72)	50.7 (27.6 to 66.5)	115 (87 to 212)
Other respiratory and intrathoracic organs	2	29/1117 (27.3)	10/726 (19.1)	1.11 (0.48 to 2.59)	−11.1 (−158.8 to 52.3)	NB (141 to NB)
Bone/mesothelial/soft tissue	4	63/2590 (25.4)	22/1540 (19.4)	1.01 (0.57 to 1.79)	−1.4 (−79.3 to 42.7)	NB (186 to NB)
Breast	4	93/6094 (14.7)	23/3911 (7.6)	0.55 (0.32 to 0.97)	44.7 (3.2 to 68.4)	305 (199 to 4225)
Gynecological	4	37/2213 (17.0)	14/1276 (14.5)	1.35 (0.64 to 2.86)	−35.4 (−186.1 to 36.0)	NB (329 to NB)
Prostate	4	203/9094 (21.6)	77/6671 (15.3)	0.81 (0.60 to 1.10)	18.7 (−9.9 to 39.8)	499 (234 to NB)
Urinary tract	4	176/6268 (27.9)	49/4099 (15.9)	0.66 (0.46 to 0.96)	33.5 (4.3 to 53.8)	216 (134 to 1699)
Central nervous system	3	50/1512 (36.3)	11/704 (21.8)	0.81 (0.38 to 1.73)	19.1 (−73.3 to 62.2)	293 (89 to NB)
Endocrine glands	4	69/2680 (26.2)	24/1541 (21.2)	0.76 (0.44 to 1.32)	23.5 (−32.1 to 55.7)	327 (138 to NB)
Hematologic malignant neoplasms	4	432/15 237 (27.7)	124/9920 (16.8)	0.68 (0.54 to 0.85)	32.3 (14.7 to 46.3)	226 (157 to 499)
Leukemia	4	183/6616 (27.1)	50/4059 (16.4)	0.70 (0.48 to 1.02)	29.9 (−2.0 to 51.8)	249 (143 to NB)
Lymphoma	4	180/5206 (33.9)	56/3469 (21.8)	0.84 (0.59 to 1.19)	16.2 (−19.4 to 41.2)	369 (145 to NB)
Myeloma	4	99/3430 (27.8)	34/2516 (18.3)	0.72 (0.46 to 1.12)	28.4 (−12.0 to 54.2)	255 (133 to NB)
Myelodysplastic syndromes	2	44/1405 (32.5)	13/930 (19.5)	0.59 (0.29 to 1.19)	41.3 (−18.5 to 70.9)	151 (87 to NB)
All cancer medications						
Cytotoxic chemotherapy	4	470/19 380 (26.1)	95/10 035 (13.0)	0.67 (0.52 to 0.87)	32.6 (12.9 to 47.9)	237 (161 to 604)
Immunotherapy	4	992/42 833 (22.7)	294/27 466 (14.2)	0.69 (0.59 to 0.80)	31.0 (19.8 to 40.7)	286 (218 to 448)
Systemic corticosteroids	4	808/27 474 (30.4)	211/16 022 (17.9)	0.70 (0.58 to 0.83)	30.5 (17.0 to 41.7)	218 (159 to 391)
Solid malignant neoplasm medications						
Cytotoxic chemotherapy	4	397/17 220 (24.9)	83/8862 (12.9)	0.69 (0.53 to 0.92)	30.5 (8.3 to 47.4)	265 (170 to 973)
Immunotherapy	4	810/35 660 (22.4)	241/22 954 (13.9)	0.69 (0.58 to 0.81)	31.2 (18.8 to 41.7)	289 (216 to 481)
Systemic corticosteroids	4	683/23 840 (29.9)	174/13 846 (17.1)	0.67 (0.55 to 0.81)	33.0 (18.6 to 44.8)	205 (151 to 364)
Hematologic malignant neoplasm medications and transplants						
Cytotoxic chemotherapy	4	146/4266 (35.9)	31/2414 (18.0)	0.73 (0.47 to 1.14)	26.5 (−14.2 to 52.7)	213 (107 to NB)
Immunotherapy	4	405/13 284 (29.6)	117/8801 (17.8)	0.67 (0.53 to 0.85)	33.0 (15.0 to 47.2)	207 (144 to 456)
Systemic corticosteroids	4	286/7272 (39.7)	79/4618 (23.6)	0.73 (0.55 to 0.98)	26.5 (2.2 to 44.8)	193 (114 to 2346)
Hematopoietic stem cell transplant	4	44/1681 (25.4)	9/1166 (10.8)	0.65 (0.28 to 1.55)	34.6 (−55.3 to 72.5)	229 (109 to NB)
Nonimmunocompromised persons						
All	4	9143/4 838 493 (1.8)	1937/1 569 552 (1.6)	0.70 (0.66 to 0.74)	30.1 (25.9 to 34.0)	3626 (3207 to 4208)
Age, y						
<65	4	3126/3 482 574 (0.9)	414/844 131 (0.7)	0.82 (0.73 to 0.92)	17.9 (7.7 to 26.9)	12 607 (8377 to 29 143)
≥65	4	6017/1 355 919 (4.1)	1523/725 421 (2.7)	0.65 (0.61 to 0.70)	34.6 (30.1 to 38.8)	1401 (1249 to 1610)

Abbreviations: HR, hazard ratio; NB, no benefit.

^a^ Pooled results from 4 health care systems.

^b^ See eTables 4 and 5 in Supplement 1 for definitions. Patients may have had more than 1 diagnosis and/or received more than 1 type of medication and were included in the analysis of each stratum in which they qualified.

^c^ Each site ran a Cox proportional hazards model on their own data, with bivalent vaccination as a time-dependent variable and adjusting for age, sex, race and ethnicity, Charlson Comorbidity Index score, and vaccination status as of the index date (September 1, 2022). Pooled HRs were calculated from the inverse variance–weighted fixed effects of the proportional hazards model results from each site.^18^

^d^ Other gastrointestinal cancers included esophageal, pancreatic, gall bladder, anus, anal canal, and anorectum.

Among persons with cancer, the rate of diagnosed COVID-19 was 112.0 per 1000 person-years for patients who received a bivalent vaccine and 116.8 per 1000 person-years for patients who did not; the adjusted VE was not significant (2.9% [95% CI, −2.6% to 8.2%) for preventing diagnosed COVID-19 (eTable 9 and eFigure 1 in Supplement 1). Similarly, the vaccine was not effective among persons with solid malignant neoplasms, hematologic malignant neoplasms, or nonimmunocompromised persons for this outcome. The rate of COVID-19–related ICU admission among persons with cancer was 3.3 per 1000 person-years among patients who received a bivalent vaccine and 5.4 per 1000 person-years among patients who did not, for a VE of 30.1% (95% CI, 7.7%-47.0%) and an NNV of 1238 (95% CI, 792-4809) to prevent 1 ICU admission (eTable 10 and eFigure 2 in Supplement 1). Although the VE among nonimmunocompromised persons to prevent COVID-19–related ICU admission was 25.5% (95% CI, 15.3%-34.4%), the NNV was 21 137 (95% CI, 15 654-35 106), as the rate of COVID-19–related ICU admission among nonimmunocompromised persons was low (0.3/1000 person-years among patients who received a bivalent vaccine vs 0.4/1000 person-years among patients who did not).

## Discussion

Persons with cancer are at increased risk of severe COVID-19, and prior studies suggest that COVID-19 vaccines provide protection against severe COVID-19. In this large study of persons with cancer with 115 033 person-years of follow-up over 2 different vaccination periods, findings are reported with substantial clinical and public health implications for patients with cancer. First, despite widespread availability of COVID-19 vaccines, vaccine uptake was relatively low, as only 69% of persons with cancer received the recommended monovalent COVID-19 vaccine booster and only 38% received a bivalent COVID-19 vaccine. Second, the overall VE for prevention of COVID-19 hospitalization for the monovalent booster (29.2%) and the bivalent vaccine (29.9%) were similar, with low NNV (166 for monovalent booster; 310 for bivalent vaccine). Third, although overall VE among persons with cancer compared with nonimmunocompromised persons was either lower (29.2% vs 50.8% for the monovalent booster, respectively) or similar (29.9% vs 30.1% for the bivalent vaccine, respectively) the NNV to prevent 1 COVID-19 hospitalization was substantially lower (166 for persons with cancer vs 1107 for nonimmunocompromised persons for the monovalent booster; 310 vs 3626 for the bivalent vaccine), due to substantially higher baseline rates of COVID-19 hospitalization among persons with cancer.

COVID-19 vaccine uptake among persons with cancer was higher than among nonimmunocompromised persons (69% vs 49% for monovalent booster; 38% vs 24% for bivalent vaccine), but for both groups, the absolute level was lower during the bivalent period. COVID-19 vaccine uptake in this study was higher than what is publicly reported for the general population, but few studies comprehensively assessed vaccine uptake in persons with cancer.^27,28^ Given the VE against severe COVID-19 illness and the favorable NNV reported herein, interventions to improve vaccine uptake in this high-risk population are urgently needed. Successful interventions to increase vaccine uptake among persons with cancer include implementation of structured vaccination protocols in oncology clinics that include electronic reminders to identify eligible patients, standing orders, and administration of vaccines by nursing staff during cancer care encounters.^29^

This is the largest epidemiologic study of COVID-19 booster VE in patients with cancer, a high-risk population of critical importance and uncertain immunologic function. COVID-19 VE is challenging to evaluate, as measured VE is dependent on person-level immunologic factors, including the host’s ability to generate neutralizing antibodies after vaccination, the vaccine formulation the individual receives, the dominant circulating SARS-CoV-2 variant at the time of vaccination, and levels of preexisting immunity in the population (which affects community exposure to the virus). Although many studies have documented COVID-19 VE in other settings, this study adds substantially to the existing understanding of COVID-19 VE by documenting VE in a large, real-world study of persons with cancer. Previously reported VE estimates for preventing COVID-19 hospitalization have ranged from 38% to 77%, which are similar to study VE estimates.^13,30,31,32^ In this study, VE and NNV changed over time due to both the protective efficacy of the vaccine as well as the changing rates of COVID-19 hospitalization, diagnosed COVID-19, and COVID-19–related ICU admission. Annual evaluations will be needed to reassess VE and NNV as vaccine formulations change, new SARS-CoV-2 variants emerge, and COVID-19 outcome rates change.

This study provides data on persons with cancer and provides stratified results by cancer type and treatment modality. Persons with cancer are a heterogenous group and thus VE might vary substantially by cancer type. The study did not find substantial variation by cancer location, noting that despite using data with 115 033 person-years of follow-up, the precision of study estimates for many cancer subgroups was low. However, study findings do suggest substantial benefit for all patients with cancer and thus support increased vaccination in all patients in this high-risk group.

Numerous questions remain about how to optimally protect persons with cancer from severe COVID-19. First, the study did not evaluate potential waning of protection after 4 to 6 months, which has been found in other studies, and thus additional doses of vaccine after 4 to 6 months as recommended are likely to be beneficial.^13,33^ Second, VE in this study was modest and thus improvements in vaccine technology are needed to improve vaccine responses and increase duration of protection in persons with cancer. Third, optimal timing of vaccination associated with cancer treatment has not been established and was not part of this study. Fourth, the mechanisms of reduced antibody responses in patients with cancer still need to be elucidated.

### Limitations

This study has limitations. First, as an observational, retrospective cohort study based on electronic health record data, there is a potential for residual confounding as well as misclassification bias of exposure if not all vaccine doses were recorded. Second, there may be underlying heterogeneity among sites that could not be addressed (eFigure 3 in Supplement 1). Because only 4 sites were included, traditional heterogeneity statistics may be biased.^24^ Additionally, the health care systems included in this study serve specific geographic areas and have underlying differences and thus might not be generalizable to all patients in the US. Third, although more than 70 000 persons were included, the absolute sample size for some cancer subtypes was small. Fourth, timing of vaccination relative to receipt of treatment was not assessed and analyses stratified by specific cancer treatments were not conducted. Fifth, COVID-19 diagnosis may be underreported given the increasing availability of at-home tests for which results may not be reported to the health care systems. However, this is unlikely to affect COVID-19 hospitalization or ICU admission.

## Conclusions

In this large study summarizing real-world experiences across 4 health care systems, substantial benefit of COVID-19 vaccination with favorable NNV to prevent severe COVID-19 in persons with cancer was found. Uptake of COVID-19 vaccine boosters was relatively low and interventions are therefore justified to increase COVID-19 vaccine uptake in this high-risk population.

## Notes

Supplement 1. eTable 1. Description of each health care system eTable 2. Characteristics of monovalent COVID-19 vaccine booster period (January 1, 2022 to August 31, 2022) patients with cancer at four health care systems eTable 3. Characteristics of bivalent COVID-19 vaccine booster period (September 1, 2022 to August 31, 2023) patients with cancer at four health care systems eTable 4. International Classification of Diseases (ICD) diagnosis codes for inclusion in study eTable 5. Medications for inclusion in the study eTable 6. International Classification of Diseases (ICD) diagnosis codes used to identify COVID-19 infection and possible COVID-19 hospitalization eTable 7. Effectiveness of one additional dose of monovalent COVID-19 vaccination (booster) compared to primary series only to prevent diagnosed COVID-19 in people with cancer and non-immunocompromised persons from January 1, 2022 to August 31, 2022, pooled results from four health care systems eTable 8. Effectiveness of one additional dose of monovalent COVID-19 vaccination (booster) compared to primary series only to prevent COVID-19 hospitalization with ICU admission in people with cancer and non-immunocompromised persons from January 1, 2022 to August 31, 2022, pooled results from four health care systems eTable 9. Effectiveness of bivalent COVID-19 vaccine compared to no bivalent vaccine to prevent diagnosed COVID-19 in people with cancer and non-immunocompromised persons from September 1, 2022 to August 31, 2023, pooled results from four health care systems eTable 10. Effectiveness of bivalent COVID-19 vaccine compared to no bivalent vaccine to prevent COVID-19 hospitalization with ICU admission in people with cancer and non-immunocompromised persons from September 1, 2022 to August 31, 2023, pooled results from four health care systems eFigure 1. Effectiveness and number needed to vaccinate to prevent diagnosed COVID-19 in people with cancer and non-immunocompromised persons: One additional dose of monovalent COVID-19 vaccination (booster) compared to primary series only (January 1, 2022 to August 31, 2022) and bivalent COVID-19 vaccine compared to no bivalent vaccine (September 1, 2022 to August 31, 2023), pooled results from four health care systems eFigure 2. Effectiveness and number needed to vaccinate to prevent COVID-19 hospitalization with ICU admission in people with cancer and non-immunocompromised persons: One additional dose of monovalent COVID-19 vaccination (booster) compared to primary series only (January 1, 2022 to August 31, 2022) and bivalent COVID-19 vaccine compared to no bivalent vaccine (September 1, 2022 to August 31, 2023), pooled results from four health care systems eFigure 3. COVID-19 vaccine effectiveness in people with cancer for prevention of COVID-19 hospitalization, diagnosed COVID-19 and COVID-19 hospitalization with intensive care unit (ICU) admission: One additional dose of monovalent COVID-19 vaccination (booster) compared to primary series only (January 1, 2022 to August 31, 2022) and bivalent COVID-19 vaccine compared to no bivalent vaccine (September 1, 2022 to August 31, 2023), site-specific results and summary results from four health care systems

Supplement 2. Data Sharing Statement
