# Supplementary material for: COVID-19 Vaccine Booster Uptake and Effectiveness Among US Adults With Cancer
Source: JAMA Oncol. 2025 Jul 17;11(9):999–1010. doi: 10.1001/jamaoncol.2025.2020 (PMC12272354; doi:10.1001/jamaoncol.2025.2020)
Supplement: Supplement 1. — eTable 1. Description of each health care system eTable 2. Characteristics of monovalent COVID-19 vaccine booster period (January 1, 2022 to August 31, 2022) patients with cancer at four health care systems eTable 3. Characteristics of bivalent COVID-19 vaccine booster period (September 1, 2022 to August 31, 2023) patients with cancer at four health care systems eTable 4. International Classification of Diseases (ICD) diagnosis codes for inclusion in study eTable 5. Medications for inclusion in the study eTable 6. International Classification of Diseases (ICD) diagnosis codes used to identify COVID-19 infection and possible COVID-19 hospitalization eTable 7. Effectiveness of one additional dose of monovalent COVID-19 vaccination (booster) compared to primary series only to prevent diagnosed COVID-19 in people with cancer and non-immunocompromised persons from January 1, 2022 to August 31, 2022, pooled results from four health care systems eTable 8. Effectiveness of one additional dose of monovalent COVID-19 vaccination (booster) compared to primary series only to prevent COVID-19 hospitalization with ICU admission in people with cancer and non-immunocompromised persons from January 1, 2022 to August 31, 2022, pooled results from four health care systems eTable 9. Effectiveness of bivalent COVID-19 vaccine compared to no bivalent vaccine to prevent diagnosed COVID-19 in people with cancer and non-immunocompromised persons from September 1, 2022 to August 31, 2023, pooled results from four health care systems eTable 10. Effectiveness of bivalent COVID-19 vaccine compared to no bivalent vaccine to prevent COVID-19 hospitalization with ICU admission in people with cancer and non-immunocompromised persons from September 1, 2022 to August 31, 2023, pooled results from four health care systems eFigure 1. Effectiveness and number needed to vaccinate to prevent diagnosed COVID-19 in people with cancer and non-immunocompromised persons: One additional dose of monovalent COVID [file jamaoncol-e252020-s001.pdf]

Supplemental Online Content

Skarbinski J, Elkin EP, Ziemba YC, et al. COVID-19 vaccine booster uptake and effectiveness among US adults with cancer. *JAMA Oncol*. Published online July 17, 2025. doi:10.1001/jamaoncol.2025.2020

eTable 1. Description of each health care system
eTable 2. Characteristics of monovalent COVID-19 vaccine booster period (January 1, 2022 to August 31, 2022) patients with cancer at four health care systems
eTable 3. Characteristics of bivalent COVID-19 vaccine booster period (September 1, 2022 to August 31, 2023) patients with cancer at four health care systems
eTable 4. International Classification of Diseases (ICD) diagnosis codes for inclusion in study
eTable 5. Medications* for inclusion in the study
eTable 6. International Classification of Diseases (ICD) diagnosis codes used to identify COVID-19 infection and possible COVID-19 hospitalization*
eTable 7. Effectiveness of one additional dose of monovalent COVID-19 vaccination (booster) compared to primary series only to prevent diagnosed COVID-19 in people with cancer and non-immunocompromised persons from January 1, 2022 to August 31, 2022, pooled results from four health care systems
eTable 8. Effectiveness of one additional dose of monovalent COVID-19 vaccination (booster) compared to primary series only to prevent COVID-19 hospitalization with ICU admission in people with cancer and non-immunocompromised persons from January 1, 2022 to August 31, 2022, pooled results from four health care systems
eTable 9. Effectiveness of bivalent COVID-19 vaccine compared to no bivalent vaccine to prevent diagnosed COVID-19 in people with cancer and non-immunocompromised persons from September 1, 2022 to August 31, 2023, pooled results from four health care systems
eTable 10. Effectiveness of bivalent COVID-19 vaccine compared to no bivalent vaccine to prevent COVID-19 hospitalization with ICU admission in people with cancer and non-immunocompromised persons from September 1, 2022 to August 31, 2023, pooled results from four health care systems
eFigure 1. Effectiveness and number needed to vaccinate to prevent diagnosed COVID-19 in people with cancer and non-immunocompromised persons: One additional dose of monovalent COVID-19 vaccination (booster) compared to primary series only (January 1, 2022 to August 31, 2022) and bivalent COVID-19 vaccine compared to no bivalent vaccine (September 1, 2022 to August 31, 2023), pooled results from four health care systems
eFigure 2. Effectiveness and number needed to vaccinate to prevent COVID-19 hospitalization with ICU admission in people with cancer and non-immunocompromised persons: One additional dose of monovalent COVID-19 vaccination (booster) compared to primary series only (January 1, 2022 to August 31, 2022) and bivalent COVID-19 vaccine compared to no bivalent vaccine (September 1, 2022 to August 31, 2023), pooled results from four health care systems
eFigure 3. COVID-19 vaccine effectiveness in people with cancer for prevention of COVID-19 hospitalization, diagnosed COVID-19 and COVID-19 hospitalization with intensive care unit (ICU) admission: One additional dose of monovalent COVID-19 vaccination (booster) compared to primary series only (January 1, 2022 to August 31, 2022) and bivalent COVID-19 vaccine compared to no bivalent vaccine (September 1, 2022 to August 31, 2023), site-specific results and summary results from four health care systems

This supplemental material has been provided by the authors to give readers additional information about their work.

© 2025 American Medical Association. All rights reserved, including those for text and data mining, AI training, and similar technologies.

Table of Contents

eTable 1. Description of each health care system 3

eTable 2. Characteristics of monovalent COVID-19 vaccine booster period (January 1, 2022 to August 31, 2022) patients with cancer at four health care systems 4

eTable 3. Characteristics of bivalent COVID-19 vaccine booster period (September 1, 2022 to August 31, 2023) patients with cancer at four health care systems 6

eTable 4. International Classification of Diseases (ICD) diagnosis codes for inclusion in study 8

eTable 5. Medications* for inclusion in the study 10

eTable 6. International Classification of Diseases (ICD) diagnosis codes used to identify COVID-19 infection and possible COVID-19 hospitalization* 12

eTable 7. Effectiveness of one additional dose of monovalent COVID-19 vaccination (booster) compared to primary series only to prevent diagnosed COVID-19 in people with cancer and non-immunocompromised persons from January 1, 2022 to August 31, 2022, pooled results from four health care systems 13

eTable 8. Effectiveness of one additional dose of monovalent COVID-19 vaccination (booster) compared to primary series only to prevent COVID-19 hospitalization with ICU admission in people with cancer and non-immunocompromised persons from January 1, 2022 to August 31, 2022, pooled results from four health care systems 15

eTable 9. Effectiveness of bivalent COVID-19 vaccine compared to no bivalent vaccine to prevent diagnosed COVID-19 in people with cancer and non-immunocompromised persons from September 1, 2022 to August 31, 2023, pooled results from four health care systems 17

eTable 10. Effectiveness of bivalent COVID-19 vaccine compared to no bivalent vaccine to prevent COVID-19 hospitalization with ICU admission in people with cancer and non-immunocompromised persons from September 1, 2022 to August 31, 2023, pooled results from four health care systems 19

eFigure 1. Effectiveness and number needed to vaccinate to prevent diagnosed COVID-19 in people with cancer and non-immunocompromised persons: One additional dose of monovalent COVID-19 vaccination (booster) compared to primary series only (January 1, 2022 to August 31, 2022) and bivalent COVID-19 vaccine compared to no bivalent vaccine (September 1, 2022 to August 31, 2023), pooled results from four health care systems 21

eFigure 2. Effectiveness and number needed to vaccinate to prevent COVID-19 hospitalization with ICU admission in people with cancer and non-immunocompromised persons: One additional dose of monovalent COVID-19 vaccination (booster) compared to primary series only (January 1, 2022 to August 31, 2022) and bivalent COVID-19 vaccine compared to no bivalent vaccine (September 1, 2022 to August 31, 2023), pooled results from four health care systems 23

eFigure 3. COVID-19 vaccine effectiveness in people with cancer for prevention of COVID-19 hospitalization, diagnosed COVID-19 and COVID-19 hospitalization with intensive care unit (ICU) admission: One additional dose of monovalent COVID-19 vaccination (booster) compared to primary series only (January 1, 2022 to August 31, 2022) and bivalent COVID-19 vaccine compared to no bivalent vaccine (September 1, 2022 to August 31, 2023), site-specific results and summary results from four health care systems 25

© 2025 American Medical Association. All rights reserved, including those for text and data mining, AI training, and similar technologies.

eTable 1. Description of each health care system

	Cedars-Sinai Health System	Kaiser Permanente Northern California	Northwell Health	Veterans Health Administration
**Geographic area**	Southern California	Northern California	New York City metropolitan area	Nationwide
**Description of health system**	Nonprofit, tertiary care hospital and multi-specialty academic health science center	Member-based health insurance; capitated largely closed integrated health system;<br>almost all services provided within network	Large health system of hospitals & physician's practices; laboratory services for Northwell Health and non-Northwell Health practices	Eligibility based on prior military service; most services provided with some exceptions; many obtain health care at both Veterans Health Administration and non-Veterans Health Administration providers.
**Health system resources**	3,000 physicians<br>5 hospitals and 36 outpatient locations	10,0000 physicians<br>21 hospitals and 203 outpatient locations	12,000 physicians<br>22 hospitals and 120 outpatient locations	26,000 physicians<br>170 hospitals and 1,300 outpatient locations
**Adult patient population**	1 million	3.7 million	6.9 million	8.7 million
**Source of vaccination data**	1) Vaccinations given within health system<br>2) Outside vaccinations recorded in electronic health record<br>3) Data feed from California Immunization Registry	1) Vaccinations given at within the health system<br>2) Outside vaccinations provided by retail pharmacies<br>3) Data feed from California Immunization Registry<br>4) Self-report	1) Vaccinations given at within the health system<br>2) Outside vaccinations recorded in electronic health record<br>3) Self-report	1) Vaccinations given at within the health system<br>2) Outside vaccinations recorded in electronic health record<br>3) Self-report<br><br>Note to be included in this analysis we required documentation of either vaccine receipt or vaccine decline in the electronic health record for inclusion due to high rate of dual users of Veterans Health Administration and non-Veterans Health Administration care

© 2025 American Medical Association. All rights reserved, including those for text and data mining, AI training, and similar technologies.

eTable 2. Characteristics of monovalent COVID-19 vaccine booster period (January 1, 2022 to August 31, 2022) patients with cancer at four health care systems

		Cedars-Sinai Health System N (%)	Northwell Health N (%)	Veterans Health Administration N (%)	Kaiser Permanente Northern California N (%)
Total N		3,232	3,177	47,623	18,799
Age (years)	18-49	535 (17)	320 (10)	1,626 (3)	2,295 (12)
	50-64	902 (28)	891 (28)	8,102 (17)	5,385 (29)
	65-74	978 (30)	1,037 (33)	20,918 (44)	6,140 (33)
	75-89	817 (25)	929 (29)	16,977 (36)	4,979 (26)
Female	Yes	1,702 (53)	1,990 (63)	3,212 (7)	10,834 (58)
	No	1,530 (47)	1,187 (37)	44,411 (93)	7,965 (42)
Race and ethnicity	Hispanic	495 (15)	282 (9)	2,646 (6)	2,698 (14)
	Black	313 (10)	422 (13)	8,950 (19)	1,500 (8)
	Asian/Pacific Islander	300 (9)	247 (8)	623 (1)	3,494 (19)
	White	1,922 (59)	1,901 (60)	32,770 (69)	10,686 (57)
	Other/Unknown	202 (6)	325 (10)	2,634 (6)	421 (2)
Charlson comorbidity index >=3	Yes	1,278 (40)	2,050 (65)	36,595 (77)	15,704 (84)
	No	1,954 (60)	1,127 (35)	11,028 (23)	3,095 (16)
Vaccination status*	Primary series only	1,072 (33)	1,916 (60)	15,003 (32)	4,575 (24)
	Primary +1 additional dose	2,160 (67)	1,261 (40)	32,620 (68)	14,224 (76)
Cancer types†	Lip/oral cavity/pharynx	167 (5)	100 (3)	2,485 (5)	520 (3)
	Colorectal	285 (9)	337 (11)	4,032 (8)	1,769 (9)
	Other gastrointestinal	436 (13)	275 (9)	5,414 (11)	1,928 (10)
	Lung	301 (9)	385 (12)	6,298 (13)	1,880 (10)
	Other respiratory and intrathoracic organs	32 (1)	44 (1)	1,512 (3)	217 (1)
	Bone/mesothelial/soft tissue	176 (5)	156 (5)	2,120 (4)	882 (5)
	Breast	853 (26)	764 (24)	1,172 (2)	4,444 (24)
	Gynecological	255 (8)	409 (13)	357 (1)	1,497 (8)
	Prostate	232 (7)	235 (7)	12,261 (26)	1,260 (7)
	Urinary tract	233 (7)	303 (10)	6,639 (14)	1,616 (9)
	Central nervous system	124 (4)	99 (3)	1,242 (3)	518 (3)
	Endocrine glands	159 (5)	133 (4)	2,484 (5)	629 (3)
	Leukemia	266 (8)	282 (9)	6,682 (14)	2,018 (11)

© 2025 American Medical Association. All rights reserved, including those for text and data mining, AI training, and similar technologies.

		Cedars-Sinai Health System N (%)	Northwell Health N (%)	Veterans Health Administration N (%)	Kaiser Permanente Northern California N (%)
	Lymphoma	295 (9)	314 (10)	4,779 (10)	2,015 (11)
	Myeloma	334 (10)	195 (6)	3,341 (7)	1,177 (6)
	Myelodysplastic syndromes	105 (3)	74 (2)	1,723 (4)	353 (2)

* As of the index date (January 1, 2022). Primary series is one dose of Janssen/J&J or two doses of Pfizer (at least 14 days apart) or Moderna (at least 21 days apart). Additional dose after the primary series must occur at least 28 days after the last dose of the primary series.

† Patients may have more than one type of cancer and are included in the analysis of each they qualify for. Therefore, counts will not sum to total N and percentages will not sum to 100.

© 2025 American Medical Association. All rights reserved, including those for text and data mining, AI training, and similar technologies.

eTable 3. Characteristics of bivalent COVID-19 vaccine booster period (September 1, 2022 to August 31, 2023) patients with cancer at four health care systems

		Cedars-Sinai Health System N (%)	Northwell Health N (%)	Veterans Health Administration N (%)	Kaiser Permanente Northern California N (%)
Total N		6,673	7,604	53,356	20,784
Age (years)	18-49	980 (15)	927 (12)	2,270 (4)	2,668 (13)
	50-64	1,751 (26)	2,151 (28)	9,179 (17)	5,856 (28)
	65-74	2,078 (31)	2,335 (31)	20,768 (39)	6,732 (32)
	75-89	1864 (28)	2,191 (29)	21,139 (40)	5,528 (27)
Female	Yes	3,995 (60)	4,635 (61)	3,996 (7)	11,963 (58)
	No	2,678 (40)	2,969 (39)	49,360 (93)	8,821 (42)
Race and ethnicity	Hispanic	922 (14)	778 (10)	2,930 (6)	3,055 (15)
	Black	588 (9)	1,054 (14)	9,852 (18)	1,699 (8)
	Asian/Pacific Islander	656 (10)	548 (7)	697 (1)	3,776 (18)
	White	4,092 (61)	4,407 (58)	36,628 (69)	11,821 (57)
	Other/Unknown	415 (6)	817 (11)	3,249 (6)	433 (2)
Charlson comorbidity index >=3	Yes	2,419 (36)	4,583 (60)	40,636 (76)	17,284 (83)
	No	4,254 (64)	3,021 (40)	12,720 (24)	3,500 (17)
Vaccination status*	None/Incomplete	928 (14)	3,586 (47)	6,154 (12)	1,438 (7)
	Primary series only	935 (14)	1,702 (22)	9,211 (17)	2,252 (11)
	Primary +1 additional dose	1,766 (26)	1,703 (22)	20,401 (38)	7,131 (34)
	Primary +2 or 3 additional doses	3,044 (46)	613 (8)	17,590 (33)	9,963 (48)
Received bivalent booster	Yes	2,383 (36)	721 (10)	20,052 (38)	10,234 (49)
	No	4,290 (64)	6,883 (90)	33,304 (62)	10,550 (51)
Cancer types†	Lip/oral cavity/pharynx	343 (5)	265 (3)	2,816 (5)	584 (3)
	Colorectal	578 (9)	795 (10)	4,674 (9)	1,992 (10)
	Other gastrointestinal	934 (14)	699 (9)	6,042 (11)	2,076 (10)
	Lung	533 (8)	876 (12)	6,877 (13)	2,047 (10)
	Other respiratory and intrathoracic organs	63 (1)	113 (1)	1,605 (3)	238 (1)

© 2025 American Medical Association. All rights reserved, including those for text and data mining, AI training, and similar technologies.

		Cedars-Sinai Health System N (%)	Northwell Health N (%)	Veterans Health Administration N (%)	Kaiser Permanente Northern California N (%)
	Bone/mesothelial/soft tissue	375 (6)	360 (5)	2,373 (4)	1,022 (5)
	Breast	1,772 (27)	1,800 (24)	1,461 (3)	4,972 (24)
	Gynecological	538 (8)	859 (11)	454 (1)	1,638 (8)
	Prostate	435 (7)	538 (7)	13,356 (25)	1,436 (7)
	Urinary tract	489 (7)	735 (10)	7,362 (14)	1,781 (9)
	Central nervous system	210 (3)	236 (3)	1,441 (3)	539 (3)
	Endocrine glands	352 (5)	300 (4)	2,861 (5)	708 (3)
	Leukemia	526 (8)	713 (9)	7,164 (13)	2,272 (11)
	Lymphoma	589 (9)	767 (10)	5,132 (10)	2,187 (11)
	Myeloma	668 (10)	486 (6)	3,518 (7)	1,274 (6)
	Myelodysplastic syndromes	217 (3)	170 (2)	1,939 (4)	396 (2)

* As of the index date (September 1, 2022). Primary series is one dose of Janssen/J&J or two doses of Pfizer (at least 14 days apart) or Moderna (at least 21 days apart). Additional doses after the primary series must occur at least 28 days after a prior dose. Incomplete means the person received only one dose of Pfizer or Moderna.

† Patients may have more than one type of cancer and are included in the analysis of each they qualify for. Therefore, counts will not sum to total N and percentages will not sum to 100.

© 2025 American Medical Association. All rights reserved, including those for text and data mining, AI training, and similar technologies.

eTable 4. International Classification of Diseases (ICD) diagnosis codes for inclusion in study

Cancer types*	ICD-10†	ICD-9†
All cancer (solid or hematologic)		
Solid malignancy		
Lip/oral cavity/pharynx	C00-C14	140-149
Colorectal	C18-C21	153-154
Other gastrointestinal	C15-C17, C22-C26	150-152, 155-157, 159
Lung	C34	162
Other respiratory and intrathoracic organs	C30-C33, C37-C39	160-161, 163-165
Bone/mesothelial/soft tissue	C40-C41, C45-C49	158, 170-171, 176
Breast	C50	174-175
Gynecological	C51-C58	179-184
Prostate	C61	185
Other male urological	C60, C62-C63	186-187
Urinary tract	C64-C68	188-189
Central nervous system	C69-C72	190-192
Endocrine glands	C73-C75, C7A-C7B, D3A	193-194, 209
Unspecified‡ solid malignancy	C76-C80, Z51.0, Z51.1	195-199, V58.1
Hematologic malignancy/condition		
Leukemia	C91-C95	204-208
Lymphoma	C81-C86, C88	200-202, 273.3
Myeloma	C90	203
Myelodysplastic syndromes	D46, D47.4, D75.81	238.72-238.75, 289.83
Unspecified‡ hematologic malignancy or other condition	C96, D47.Z2, D47.Z9, D59.5, D61.0, D61.2, D61.9	238.79, 284
Hematologic transplant	D89.81, M31.11, T86.0, T86.5, Z48.290, Z94.81, Z94.84	279.5, 996.85, 996.88, V42.81-V42.82
Non-immunocompromised	None of the above and none of the following:<br><br>Solid organ transplant: T86.1-T86.4, T86.81, T86.85, Z48.21-Z48.24, Z48.280, Z94.0-Z94.4, Z94.82-Z94.83<br><br>Autoimmune/inflammatory: D86, E85, G35-G36, G37.1, G37.3, G37.8, G37.9, G61.0, G61.9, K50-K51, L10, L40.0, L40.5, L93.0, L93.2, L94, M04-M08, M30, M31.3, M31.5, M31.7, M32-M34, M35.0, M35.3, M35.81, M45-M46<br><br>Other immunodeficiency conditions: D70.0, D71, D72.0, D80-D84, D89.0-D89.4, D89.82-D89.89, D89.9	None of the above and none of the following:<br><br>Solid organ transplant: 996.81-996.84, 996.86-996.87, V42.0-V42.1, V42.6-V42.7, V42.83-V42.84<br><br>Autoimmune/inflammatory: 136.0, 135, 277.3, 340, 341.0, 341.2, 341.8-341.9, 357.0, 357.9, 446.0-446.1, 446.4-446.5, 555-556, 694.4, 695.4, 696.0-696.2, 701.0, 709.3, 710.0-710.4, 710.8-710.9, 713.1, 714, 720, 725<br><br>Other immunodeficiency conditions: 273.0-273.2, 277.6, 279.0-279.4, 279.8-279.9, 288.01, 288.1-288.2

*Any occurrence in electronic medical record from encounter diagnoses and/or problem lists (sites determined best sources for their system). Diagnosis codes recorded at any time in patient's medical record.

© 2025 American Medical Association. All rights reserved, including those for text and data mining, AI training, and similar technologies.

†All more specific billable codes that are a subset of any non-billable/non-specific codes shown in this table were included in the code list for querying diagnosis data.
‡If a patient had a specific diagnosis code in their chart for any solid or hematologic malignancy, then unspecified diagnosis codes are excluded for that patient when determining cancer type(s).

© 2025 American Medical Association. All rights reserved, including those for text and data mining, AI training, and similar technologies.

eTable 5. Medications* for inclusion in the study

Cytotoxic chemotherapy			
ado-trastuzumab	cytarabine	inotuzumab	plicamycin
altretamine	dacarbazine	irinotecan	polatuzumab
arsenic trioxide	dactinomycin	ixabepilone	porfimer
asparaginase	daunorubicin	lomustine	pralatrexate
azacitidine	decitabine	loncastuximab	procarbazine
belantamab	docetaxel	lurbinectedin	sacituzumab
bendamustine	doxorubicin	mechlorethamine	streptozocin
bleomycin	enfortumab	melphalan	temozolomide
brentuximab	epirubicin	mercaptopurine	teniposide
busulfan	eribulin	mitomycin	thioguanine
cabazitaxel	estramustine	mitotane	thiotepa
calaspargase	etoposide	mitoxantrone	tisotumab
capecitabine	fam-trastuzumab	moxetumomab	topotecan
carboplatin	floxuridine	nelarabine	trabectedin
carmustine	fludarabine	omacetaxine	trifluridine
chlorambucil	gemcitabine	oxaliplatin	trimetrexate
cisplatin	gemtuzumab	paclitaxel	uracil mustard
cladribine	hydroxyurea	pegaspargase	valrubicin
clofarabine	idarubicin	pemetrexed	vinblastine
cyclophosphamide	ifosfamide	pentostatin	vincristine
			vinorelbine
**Immunotherapy**			
abatacept	copanlisib	lapatinib	romidepsin
abemaciclib	crizotinib	larotrectinib	ropeginterferon
acalabrutinib	cyclosporine	leflunomide	rucaparib
adalimumab	dabrafenib	lenalidomide	ruxolitinib
afatinib	daclizumab	lenvatinib	sarilumab
aflibercept	dacomitinib	lisocabtagene	secukinumab
aldesleukin	daratumumab	lorlatinib	selinexor
alectinib	dasatinib	margetuximab	selpercatinib
alefacept	denileukin	methotrexate	selumetinib
alemtuzumab	denosumab	midostaurin	siltuximab
alpelisib	dinutuximab	mogamulizumab	sipuleucel
amivantamab	dostarlimab	muromonab	sirolimus
anakinra	durvalumab	mycophenolate	sonidegib
atezolizumab	duvelisib	natalizumab	sorafenib
auranofin	eculizumab	naxitamab	sotorasib
aurothioglucose	efalizumab	necitumumab	sulfasalazine
avapritinib	elotuzumab	neratinib	sunitinib
avelumab	enasidenib	nilotinib	tacrolimus
axicabtagene	encorafenib	nintedanib	tafasitamab
axitinib	entrectinib	niraparib	tagraxofusp
azathioprine	erdafitinib	nivolumab	talazoparib
baricitinib	erlotinib	obinutuzumab	talimogene
basiliximab	etanercept	ofatumumab	tazemetostat
belatacept	everolimus	olaparib	temsirolimus
belimumab	fedratinib	olaratumab	tepotinib
belinostat	fingolimod	osimertinib	thalidomide

© 2025 American Medical Association. All rights reserved, including those for text and data mining, AI training, and similar technologies.

bevacizumab<br>bexarotene<br>binimetinib<br>blinatumomab<br>bortezomib<br>bosutinib<br>brexucabtagene<br>brigatinib<br>brodalumab<br>cabozantinib<br>canakinumab<br>capmatinib<br>carfilzomib<br>cemiplimab<br>ceritinib<br>certolizumab<br>cetuximab<br>ciltacabtagene<br>cobimetinib	gefitinib<br>gilteritinib<br>glasdegib<br>glatiramer<br>gold sodium thiomalate<br>golimumab<br>guselkumab<br>ibritumomab<br>ibrutinib<br>idecabtagene<br>idelalisib<br>imatinib<br>infliximab<br>interferon<br>ipilimumab<br>isatuximab<br>ivosidenib<br>ixazomib<br>ixekizumab	palbociclib<br>panitumumab<br>panobinostat<br>pazopanib<br>pegademase<br>peginterferon<br>pembrolizumab<br>pemigatinib<br>pertuzumab<br>pexidartinib<br>pomalidomide<br>ponatinib<br>pralsetinib<br>ramucirumab<br>regorafenib<br>ribociclib<br>ripretinib<br>risankizumab<br>rituximab	tildrakizumab<br>tisagenlecleucel<br>tivozanib<br>tocilizumab<br>tofacitinib<br>tositumomab<br>trametinib<br>trastuzumab<br>tucatinib<br>umbralisib<br>upadacitinib<br>ustekinumab<br>vandetanib<br>vemurafenib<br>venetoclax<br>vismodegib<br>voclosporin<br>vorinostat<br>zanubrutinib
**Systemic† corticosteroids**			
betamethasone<br>dexamethasone<br>methylprednisolone<br>prednisolone<br>triamcinolone			

*Cancer patient must have received cytotoxic chemotherapy and/or immunotherapy during the year prior to the index date to be included in the analysis. Systemic corticosteroids may be used as additional treatment but may not be used as monotherapy. Non-immunocompromised patients could not have received any of these medications in the year prior to the index date.

†Route of medication was oral and/or intravenous.

© 2025 American Medical Association. All rights reserved, including those for text and data mining, AI training, and similar technologies.

eTable 6. International Classification of Diseases (ICD) diagnosis codes used to identify COVID-19 infection and possible COVID-19 hospitalization*

**Disease/Condition**	**ICD-10†**	**Used for outcome definitions**
COVID-19	J12.82, U07.1	Infection and hospitalization
Coronavirus infection	B34.2, B97.21, B97.29, J12.81	Infection and hospitalization
Pneumonia	J12.89, J12.9, J18	Hospitalization
Respiratory infection (other)	J20.8, J22	Hospitalization
Acute respiratory distress syndrome	J80	Hospitalization
Respiratory failure	J96.0, J96.2, J96.9	Hospitalization
Sepsis & systemic inflammation	A41, M35.8, R65.1, R65.2	Hospitalization

*See text for full definition of COVID-19 hospitalization outcome.
†All more specific billable codes that are a subset of any non-billable/non-specific codes shown in this table were included in the code list for querying diagnosis data.

© 2025 American Medical Association. All rights reserved, including those for text and data mining, AI training, and similar technologies.

eTable 7. Effectiveness of one additional dose of monovalent COVID-19 vaccination (booster) compared to primary series only to prevent diagnosed COVID-19 in people with cancer and non-immunocompromised persons from January 1, 2022 to August 31, 2022, pooled results from four health care systems

Cancer types*	# sites contributing to pooled analysis	Primary series only† # events/# people (rate per 1000 person-years)	Primary series +1 additional vaccination† # events/# people (rate per 1000 person-years)	Pooled adjusted hazard ratio‡ (95% CI)	Effectiveness of one additional vaccination (%) (95% CI)	Number needed to vaccinate (95% CI)
All cancer (solid or hematologic)	4	2,339/22,566 (241.8)	4,808/50,265 (216.3)	0.92 (0.87 - 0.96)	8.5 (3.7 - 13.0)	110 (71 - 253)
Solid malignancy	4	1,924/19,131 (234.0)	4,089/42,910 (214.8)	0.94 (0.89 - 0.99)	6.1 (0.8 - 11.2)	157 (85 - 1254)
Lip/oral cavity/pharynx	4	109/1,012 (245.7)	204/2,260 (202.0)	0.82 (0.64 - 1.04)	18.3 (-3.8 - 35.7)	50 (25 - NB)
Colorectal	4	208/2,215 (218.8)	385/4,208 (201.3)	0.94 (0.79 - 1.12)	6.3 (-11.7 - 21.3)	163 (47 - NB)
Other gastrointestinal	4	253/2,359 (267.9)	553/5,694 (231.4)	0.84 (0.73 - 0.98)	15.5 (1.6 - 27.5)	54 (30 - 547)
Lung	4	281/2,742 (257.0)	567/6,122 (212.3)	0.88 (0.76 - 1.02)	11.8 (-2.1 - 23.9)	74 (36 - NB)
Other respiratory and intrathoracic organs	2	65/541 (303.6)	85/1,188 (157.1)	0.49 (0.35 - 0.69)	50.7 (31.4 - 64.6)	14 (11 - 24)
Bone/mesothelial/soft tissue	4	114/1,004 (261.4)	211/2,330 (207.5)	0.78 (0.61 - 0.99)	22.2 (1.4 - 38.6)	39 (22 - 617)
Breast	4	226/2,199 (245.8)	493/5,034 (226.5)	0.98 (0.83 - 1.15)	2.2 (-15.2 - 16.9)	423 (54 - NB)
Gynecological	4	84/856 (230.9)	131/1,662 (183.5)	0.82 (0.61 - 1.09)	18.4 (-9.2 - 39.0)	52 (24 - NB)
Prostate	4	318/3,916 (180.1)	855/10,072 (188.0)	1.03 (0.90 - 1.17)	-2.7 (-17.0 - 9.8)	NB (124 - NB)
Urinary tract	4	250/2,534 (231.2)	611/6,257 (217.5)	0.93 (0.80 - 1.08)	7.4 (-7.5 - 20.2)	132 (47 - NB)
Central nervous system	4	68/690 (241.8)	133/1,293 (237.0)	1.02 (0.75 - 1.38)	-1.6 (-37.8 - 25.1)	NB (36 - NB)
Endocrine glands	4	116/1,086 (245.4)	222/2,319 (213.5)	0.85 (0.68 - 1.07)	14.8 (-7.5 - 32.5)	62 (27 - NB)
Hematologic malignancy	4	799/6,403 (296.3)	1,693/15,046 (262.9)	0.90 (0.82 - 0.98)	10.2 (2.2 - 17.7)	76 (44 - 367)
Leukemia	4	337/2,838 (276.0)	689/6,410 (245.8)	0.89 (0.78 - 1.02)	10.9 (-1.9 - 22.0)	76 (37 - NB)
Lymphoma	4	323/2,267 (351.0)	682/5,136 (319.4)	0.95 (0.83 - 1.09)	5.1 (-8.9 - 17.2)	136 (39 - NB)
Myeloma	4	169/1,391 (293.4)	397/3,656 (264.6)	0.85 (0.70 - 1.02)	15.3 (-1.8 - 29.5)	51 (26 - NB)
Myelodysplastic syndromes	4	84/692 (299.8)	157/1,563 (232.7)	0.80 (0.61 - 1.05)	20.0 (-5.0 - 39.1)	38 (19 - NB)
All cancer - medications						
Cytotoxic chemotherapy	4	919/8,205 (281.9)	1,551/15,662 (231.2)	0.85 (0.78 - 0.92)	15.4 (8.0 - 22.2)	52 (36 - 102)
Immunotherapy	4	1,805/17,310 (241.4)	3,950/40,532 (220.9)	0.93 (0.88 - 0.98)	7.0 (1.5 - 12.1)	134 (76 - 607)
Systemic corticosteroids	4	1,406/11,111 (318.2)	2,699/23,814 (269.3)	0.88 (0.83 - 0.94)	11.8 (5.8 - 17.5)	62 (42 - 128)
Solid malignancy – medications						
Cytotoxic chemotherapy	4	780/7,269 (267.3)	1,363/13,998 (225.9)	0.87 (0.79 - 0.95)	13.4 (5.1 - 20.9)	64 (40 - 167)
Immunotherapy	4	1,441/14,312 (232.4)	3,282/33,979 (218.1)	0.95 (0.90 - 1.02)	4.6 (-1.7 - 10.4)	213 (93 - NB)
Systemic corticosteroids	4	1,193/9,675 (307.5)	2,323/20,837 (263.3)	0.89 (0.83 - 0.95)	11.2 (4.6 - 17.3)	68 (43 - 166)
Hematologic malignancy – medications & transplants						
Cytotoxic chemotherapy	4	265/1,866 (376.4)	464/3,661 (312.9)	0.85 (0.73 - 1.00)	14.9 (0.5 - 27.2)	43 (23 - 1353)

© 2025 American Medical Association. All rights reserved, including those for text and data mining, AI training, and similar technologies.

Cancer types*	# sites contributing to pooled analysis	Primary series only† # events/# people (rate per 1000 person-years)	Primary series +1 additional vaccination† # events/# people (rate per 1000 person-years)	Pooled adjusted hazard ratio‡ (95% CI)	Effectiveness of one additional vaccination (%) (95% CI)	Number needed to vaccinate (95% CI)
Immunotherapy	4	703/5,581 (298.3)	1,549/13,333 (272.8)	0.92 (0.84 - 1.01)	8.0 (-0.8 - 16.0)	98 (48 - NB)
Systemic corticosteroids	4	457/2,986 (404.1)	964/6,818 (353.3)	0.89 (0.79 - 0.99)	11.5 (0.7 - 21.1)	53 (28 - 941)
Hematologic transplant	4	88/702 (333.2)	178/1,668 (269.1)	0.80 (0.61 - 1.04)	20.4 (-3.8 - 39.0)	34 (17 - NB)
Non-immunocompromised						
All	4	150,833/2,483,954 (132.7)	167,146/2,386,184 (131.4)	0.95 (0.94 - 0.95)	5.3 (4.6 - 6.0)	302 (267 - 348)
<65 years old	4	126,480/1,822,140 (158.7)	125,283/1,345,946 (167.8)	0.95 (0.94 - 0.96)	4.8 (4.0 - 5.6)	282 (243 - 337)
>=65 years old	4	24,353/661,814 (71.7)	41,863/1,040,238 (79.7)	0.95 (0.94 - 0.97)	4.9 (3.3 - 6.4)	592 (450 - 871)

*See supplemental tables 2 and 3 for definitions. Patients may have more than one diagnosis and/or received more than one type of medication and are included in the analysis of each stratum they qualify for.

†As of index date January 1, 2022. Primary series is one dose of Janssen/J&J or two doses of Pfizer (at least 14 days apart) or Moderna (at least 21 days apart). Additional vaccination must be at least 28 days after prior vaccination.

‡Each site ran a Cox proportional hazards model on their own data including vaccination status as of the index date (January 1, 2022) and adjusting for age, sex, race and ethnicity, and Charlson index. Pooled hazard ratios were calculated from the inverse variance-weighted fixed effects of the proportional hazards model results from each site[18].

Other gastrointestinal cancers include esophageal, pancreatic, gall bladder, anus, anal canal, and anorectum cancer

NB = no benefit

© 2025 American Medical Association. All rights reserved, including those for text and data mining, AI training, and similar technologies.

eTable 8. Effectiveness of one additional dose of monovalent COVID-19 vaccination (booster) compared to primary series only to prevent COVID-19 hospitalization with ICU admission in people with cancer and non-immunocompromised persons from January 1, 2022 to August 31, 2022, pooled results from four health care systems

Cancer types*	# sites contributing to pooled analysis	Primary series only† # events/# people (rate per 1000 person-years)	Primary series +1 additional vaccination† # events/# people (rate per 1000 person-years)	Pooled adjusted hazard ratio‡ (95% CI)	Effectiveness of one additional vaccination (%) (95% CI)	Number needed to vaccinate (95% CI)
All cancer (solid or hematologic)	4	139/22,566 (13.3)	218/50,265 (9.2)	0.64 (0.52 - 0.80)	35.6 (20.0 - 48.3)	423 (312 - 756)
Solid malignancy	4	110/19,131 (12.4)	192/42,910 (9.5)	0.72 (0.57 - 0.92)	27.9 (8.3 - 43.4)	578 (372 - 1939)
Lip/oral cavity/pharynx	1					
Colorectal	2	13/1,910 (15.3)	18/3,891 (9.7)	0.60 (0.29 - 1.24)	40.0 (-23.7 - 70.9)	328 (185 - NB)
Other gastrointestinal	3	20/2,200 (21.3)	32/5,578 (12.9)	0.56 (0.32 - 0.98)	44.1 (1.5 - 68.3)	214 (138 - 6123)
Lung	2	25/2,422 (24.7)	33/5,756 (12.5)	0.54 (0.31 - 0.91)	46.5 (8.8 - 68.6)	175 (118 - 924)
Other respiratory and intrathoracic organs	2	6/541 (25.7)	9/1,188 (16.1)	0.54 (0.19 - 1.54)	45.6 (-53.9 - 80.7)	172 (97 - NB)
Bone/mesothelial/soft tissue	2	10/844 (26.3)	14/2,158 (14.2)	0.49 (0.21 - 1.11)	51.4 (-10.7 - 78.7)	149 (97 - NB)
Breast	3	5/1,810 (6.3)	6/4,251 (3.1)	0.56 (0.16 - 1.95)	44.1 (-95.4 - 84.0)	724 (379 - NB)
Gynecological	3	3/722 (9.3)	5/1,439 (7.7)	1.26 (0.28 - 5.60)	-25.5 (-459.9 - 71.8)	NB (301 - NB)
Prostate	2	26/3,723 (14.8)	34/9,798 (7.3)	0.44 (0.26 - 0.73)	56.5 (27.1 - 74.0)	240 (183 - 502)
Urinary tract	2	12/2,291 (11.6)	41/5,964 (14.5)	1.28 (0.67 - 2.44)	-27.7 (-144.2 - 33.2)	NB (521 - NB)
Central nervous system	2	9/584 (36.6)	10/1,176 (18.4)	0.53 (0.21 - 1.33)	46.9 (-32.7 - 78.8)	118 (70 - NB)
Endocrine glands	2	9/961 (20.6)	7/2,152 (6.9)	0.28 (0.10 - 0.77)	72.2 (22.6 - 90.0)	135 (108 - 434)
Hematologic malignancy	4	59/6,403 (19.9)	93/15,046 (13.5)	0.60 (0.43 - 0.84)	40.1 (16.4 - 57.1)	252 (176 - 616)
Leukemia	2	26/2,571 (21.8)	37/6,129 (13.0)	0.52 (0.31 - 0.87)	47.8 (13.3 - 68.5)	193 (134 - 698)
Lymphoma	2	22/1,964 (25.4)	46/4,830 (21.2)	0.76 (0.45 - 1.27)	24.3 (-26.6 - 54.8)	327 (145 - NB)
Myeloma	3	14/1,266 (24.5)	22/3,586 (13.9)	0.51 (0.25 - 1.04)	48.6 (-3.8 - 74.5)	169 (110 - NB)
Myelodysplastic syndromes	2	9/602 (34.4)	11/1,474 (16.4)	0.50 (0.20 - 1.23)	50.4 (-22.7 - 80.0)	116 (73 - NB)
All cancer - medications						
Cytotoxic chemotherapy	4	54/8,205 (15.2)	69/15,662 (9.7)	0.61 (0.42 - 0.87)	39.2 (12.6 - 57.7)	338 (229 - 1053)
Immunotherapy	4	107/17,310 (13.3)	184/40,532 (9.7)	0.67 (0.53 - 0.85)	33.0 (14.5 - 47.5)	459 (319 - 1045)
Systemic corticosteroids	4	90/11,111 (18.4)	139/23,814 (12.9)	0.64 (0.49 - 0.84)	35.9 (16.0 - 51.2)	304 (213 - 685)
Solid malignancy – medications						
Cytotoxic chemotherapy	4	43/7,269 (13.6)	60/13,998 (9.4)	0.68 (0.46 - 1.02)	31.6 (-2.1 - 54.2)	468 (272 - NB)
Immunotherapy	4	83/14,312 (12.4)	161/33,979 (10.1)	0.74 (0.57 - 0.98)	25.6 (2.4 - 43.2)	631 (373 - 6620)
Systemic corticosteroids	4	73/9,675 (17.1)	122/20,837 (12.9)	0.69 (0.51 - 0.93)	31.2 (7.4 - 48.9)	377 (240 - 1595)

© 2025 American Medical Association. All rights reserved, including those for text and data mining, AI training, and similar technologies.

Cancer types*	# sites contributing to pooled analysis	Primary series only† # events/# people (rate per 1000 person-years)	Primary series +1 additional vaccination† # events/# people (rate per 1000 person-years)	Pooled adjusted hazard ratio‡ (95% CI)	Effectiveness of one additional vaccination (%) (95% CI)	Number needed to vaccinate (95% CI)
Hematologic malignancy – medications & transplants						
Cytotoxic chemotherapy	3	20/1,666 (28.8)	23/3,552 (14.8)	0.44 (0.24 - 0.82)	55.6 (18.3 - 75.9)	126 (92 - 384)
Immunotherapy	4	50/5,581 (19.3)	87/13,333 (14.3)	0.67 (0.47 - 0.96)	32.9 (4.2 - 53.0)	317 (196 - 2468)
Systemic corticosteroids	4	39/2,986 (30.4)	61/6,818 (20.4)	0.61 (0.41 - 0.92)	38.8 (7.8 - 59.4)	171 (112 - 858)
Hematologic transplant	3	5/641 (18.5)	6/1,631 (8.7)	0.54 (0.15 - 1.87)	46.4 (-87.3 - 84.7)	235 (128 - NB)
Non-immunocompromised						
All	4	972/2,483,954 (0.8)	584/2,386,184 (0.4)	0.47 (0.42 - 0.52)	53.0 (47.8 - 57.7)	4621 (4245 - 5126)
<65 years old	4	363/1,822,140 (0.4)	150/1,345,946 (0.2)	0.51 (0.42 - 0.62)	49.4 (38.4 - 58.5)	9390 (7936 - 12084)
>=65 years old	4	609/661,814 (1.8)	434/1,040,238 (0.8)	0.45 (0.40 - 0.51)	54.7 (48.7 - 60.0)	2086 (1902 - 2343)

*See supplemental tables 2 and 3 for definitions. Patients may have more than one diagnosis and/or received more than one type of medication and are included in the analysis of each stratum they qualify for.
†As of index date January 1, 2022. Primary series is one dose of Janssen/J&J or two doses of Pfizer (at least 14 days apart) or Moderna (at least 21 days apart). Additional vaccination must be at least 28 days after prior vaccination.
‡Each site ran a Cox proportional hazards model on their own data including vaccination status as of the index date (January 1, 2022) and adjusting for age, sex, race and ethnicity, and Charlson index. Pooled hazard ratios were calculated from the inverse variance-weighted fixed effects of the proportional hazards model results from each site[18].

Other gastrointestinal cancers include esophageal, pancreatic, gall bladder, anus, anal canal, and anorectum cancer

NB = no benefit

© 2025 American Medical Association. All rights reserved, including those for text and data mining, AI training, and similar technologies.

eTable 9. Effectiveness of bivalent COVID-19 vaccine compared to no bivalent vaccine to prevent diagnosed COVID-19 in people with cancer and non-immunocompromised persons from September 1, 2022 to August 31, 2023, pooled results from four health care systems

Cancer types*	# sites contributing to pooled analysis	No bivalent vaccination<br><br># events/# people (rate per 1000 person-years)	Bivalent vaccination<br><br># events/# people (rate per 1000 person-years)	Pooled adjusted hazard ratio† (95% CI)	Effectiveness of bivalent vaccination (%) (95% CI)	Number needed to vaccinate (95% CI)
All cancer (solid or hematologic)	4	6,141/54,711 (116.8)	2,644/33,706 (112.0)	0.97 (0.92 - 1.03)	2.9 (-2.6 - 8.2)	619 (222 - NB)
Solid malignancy	4	5,106/46,544 (114.7)	2,193/28,645 (109.1)	0.96 (0.90 - 1.02)	3.9 (-2.1 - 9.5)	471 (193 - NB)
Lip/oral cavity/pharynx	4	268/2,499 (112.2)	111/1,509 (107.1)	0.99 (0.76 - 1.28)	1.0 (-28.5 - 23.8)	1826 (78 - NB)
Colorectal	4	525/5,352 (105.4)	180/2,687 (96.8)	0.92 (0.75 - 1.12)	8.4 (-11.9 - 25.0)	239 (79 - NB)
Other gastrointestinal	4	680/6,209 (125.2)	276/3,542 (114.3)	0.97 (0.82 - 1.15)	3.0 (-14.8 - 18.0)	575 (94 - NB)
Lung	4	788/6,562 (139.3)	279/3,771 (108.8)	0.85 (0.73 - 1.00)	14.6 (-0.4 - 27.4)	105 (55 - NB)
Other respiratory and intrathoracic organs	3	115/1,160 (108.4)	50/746 (98.3)	0.94 (0.62 - 1.41)	6.3 (-41.5 - 38.0)	308 (50 - NB)
Bone/mesothelial/soft tissue	4	281/2,590 (118.2)	141/1,540 (133.3)	1.17 (0.91 - 1.51)	-17.2 (-50.8 - 8.9)	NB (201 - NB)
Breast	4	746/6,094 (125.2)	327/3,911 (116.5)	0.88 (0.75 - 1.04)	11.8 (-4.0 - 25.3)	143 (66 - NB)
Gynecological	4	231/2,213 (111.4)	117/1,276 (129.7)	1.04 (0.78 - 1.38)	-3.8 (-37.5 - 21.6)	NB (87 - NB)
Prostate	4	930/9,094 (103.4)	477/6,671 (100.4)	1.03 (0.90 - 1.17)	-2.9 (-17.3 - 9.7)	NB (208 - NB)
Urinary tract	4	711/6,268 (117.7)	328/4,099 (114.1)	1.01 (0.86 - 1.19)	-1.4 (-19.0 - 13.5)	NB (132 - NB)
Central nervous system	4	198/1,654 (138.5)	61/772 (116.2)	0.76 (0.54 - 1.06)	24.4 (-6.3 - 46.2)	63 (33 - NB)
Endocrine glands	4	333/2,680 (133.8)	109/1,541 (102.8)	0.74 (0.57 - 0.95)	26.2 (5.1 - 42.7)	60 (36 - 311)
Hematologic malignancy	4	2,019/15,237 (136.4)	902/9,920 (132.7)	1.03 (0.93 - 1.13)	-2.9 (-13.2 - 6.5)	NB (241 - NB)
Leukemia	4	833/6,616 (130.1)	345/4,059 (121.9)	1.01 (0.87 - 1.18)	-1.5 (-18.4 - 13.0)	NB (125 - NB)
Lymphoma	4	806/5,206 (161.9)	353/3,469 (150.7)	1.11 (0.95 - 1.29)	-10.8 (-29.2 - 5.0)	NB (268 - NB)
Myeloma	4	446/3,430 (131.7)	229/2,516 (134.1)	1.03 (0.85 - 1.25)	-3.0 (-25.2 - 15.3)	NB (105 - NB)
Myelodysplastic syndromes	3	228/1,539 (162.3)	83/1,013 (123.5)	0.74 (0.55 - 0.99)	26.3 (0.9 - 45.2)	50 (29 - 1526)
All cancer - medications						
Cytotoxic chemotherapy	4	2,291/19,380 (134.2)	795/10,035 (116.9)	0.91 (0.83 - 1.01)	8.5 (-0.9 - 17.1)	186 (92 - NB)
Immunotherapy	4	4,923/42,833 (118.2)	2,194/27,466 (113.7)	0.99 (0.93 - 1.05)	1.2 (-5.0 - 7.1)	1450 (253 - NB)
Systemic corticosteroids	4	3,801/27,474 (152.2)	1,436/16,022 (132.1)	0.94 (0.88 - 1.02)	5.6 (-1.6 - 12.3)	253 (115 - NB)
Solid malignancy – medications						
Cytotoxic chemotherapy	4	1,974/17,220 (130.8)	677/8,862 (112.5)	0.90 (0.81 - 1.00)	9.9 (-0.2 - 19.0)	164 (85 - NB)
Immunotherapy	4	3,984/35,660 (115.2)	1,789/22,954 (110.7)	0.98 (0.91 - 1.05)	2.3 (-4.5 - 8.6)	807 (212 - NB)
Systemic corticosteroids	4	3,194/23,840 (148.1)	1,208/13,846 (128.1)	0.93 (0.86 - 1.01)	7.1 (-0.6 - 14.3)	204 (101 - NB)
Hematologic malignancy – medications & transplants						
Cytotoxic chemotherapy	4	645/4,266 (169.2)	229/2,414 (145.7)	0.96 (0.80 - 1.16)	4.0 (-15.6 - 20.2)	324 (63 - NB)
Immunotherapy	4	1,804/13,284 (139.2)	816/8,801 (135.1)	1.04 (0.94 - 1.15)	-4.4 (-15.4 - 5.6)	NB (274 - NB)

© 2025 American Medical Association. All rights reserved, including those for text and data mining, AI training, and similar technologies.

Cancer types*	# sites contributing to pooled analysis	No bivalent vaccination<br><br># events/# people (rate per 1000 person-years)	Bivalent vaccination<br><br># events/# people (rate per 1000 person-years)	Pooled adjusted hazard ratio† (95% CI)	Effectiveness of bivalent vaccination (%) (95% CI)	Number needed to vaccinate (95% CI)
Systemic corticosteroids	4	1,246/7,272 (185.6)	494/4,618 (162.7)	1.00 (0.88 - 1.13)	0.3 (-13.2 - 12.2)	4018 (96 - NB)
Hematologic transplant	4	234/1,681 (143.3)	94/1,166 (122.5)	0.97 (0.73 - 1.30)	3.0 (-29.6 - 27.3)	508 (54 - NB)
Non-immunocompromised						
All	4	195,380/4,838,493 (40.0)	71,738/1,569,552 (61.9)	1.03 (1.02 - 1.04)	-3.3 (-4.4 - -2.3)	NB (NB - NB)
<65 years old	4	136,981/3,482,574 (39.7)	38,907/844,131 (64.0)	1.07 (1.06 - 1.09)	-7.3 (-8.7 - -5.9)	NB (NB - NB)
>=65 years old	4	58,399/1,355,919 (40.9)	32,831/725,421 (59.6)	0.98 (0.96 - 1.00)	1.9 (0.2 - 3.5)	2643 (1422 - 20804)

*See supplemental tables 2 and 3 for definitions. Patients may have more than one diagnosis and/or received more than one type of medication and are included in the analysis of each stratum they qualify for.

†Each site ran a Cox proportional hazards model on their own data with bivalent vaccination as a time-dependent variable and adjusting for age, sex, race and ethnicity, Charlson index, and vaccination status as of the index date (September 1, 2022). Pooled hazard ratios were calculated from the inverse variance-weighted fixed effects of the proportional hazards model results from each site[18].

Other gastrointestinal cancers include esophageal, pancreatic, gall bladder, anus, anal canal, and anorectum cancer

NB = no benefit

© 2025 American Medical Association. All rights reserved, including those for text and data mining, AI training, and similar technologies.

eTable 10. Effectiveness of bivalent COVID-19 vaccine compared to no bivalent vaccine to prevent COVID-19 hospitalization with ICU admission in people with cancer and non-immunocompromised persons from September 1, 2022 to August 31, 2023, pooled results from four health care systems

Cancer types*	# sites contributing to pooled analysis	No bivalent vaccination<br><br># events/# people (rate per 1000 person-years)	Bivalent vaccination<br><br># events/# people (rate per 1000 person-years)	Pooled adjusted hazard ratio† (95% CI)	Effectiveness of bivalent vaccination (%) (95% CI)	Number needed to vaccinate (95% CI)
All cancer (solid or hematologic)	4	299/54,711 (5.4)	84/33,706 (3.3)	0.70 (0.53 - 0.92)	30.1 (7.7 - 47.0)	1238 (792 - 4809)
Solid malignancy	4	253/46,544 (5.4)	71/28,645 (3.3)	0.70 (0.52 - 0.95)	29.9 (5.3 - 48.1)	1244 (773 - 6981)
Lip/oral cavity/pharynx	1					
Colorectal	2	19/4,215 (4.6)	4/2,451 (2.2)	0.55 (0.16 - 1.90)	45.2 (-89.8 - 84.2)	966 (518 - NB)
Other gastrointestinal	3	39/5,575 (7.5)	18/3,477 (7.1)	1.37 (0.69 - 2.72)	-37.0 (-172.1 - 31.0)	NB (857 - NB)
Lung	1					
Other respiratory and intrathoracic organs	0					
Bone/mesothelial/soft tissue	2	11/2,014 (5.6)	5/1,381 (4.9)	0.80 (0.22 - 2.87)	20.0 (-187.4 - 77.7)	1793 (460 - NB)
Breast	1					
Gynecological	1					
Prostate	2	45/8,366 (5.1)	26/6,426 (5.3)	1.03 (0.59 - 1.81)	-3.0 (-81.2 - 41.5)	NB (939 - NB)
Urinary tract	2	35/5,317 (6.4)	13/3,826 (4.5)	0.82 (0.39 - 1.72)	18.1 (-71.9 - 61.0)	1722 (510 - NB)
Central nervous system	1					
Endocrine glands	2	21/2,187 (9.7)	6/1,382 (5.9)	0.52 (0.19 - 1.41)	48.2 (-41.0 - 81.0)	430 (256 - NB)
Hematologic malignancy	4	116/15,237 (7.4)	41/9,920 (5.5)	0.84 (0.56 - 1.28)	15.5 (-27.7 - 44.1)	1756 (617 - NB)
Leukemia	2	39/5,638 (6.7)	16/3,798 (5.6)	0.82 (0.42 - 1.63)	17.7 (-62.7 - 58.3)	1708 (516 - NB)
Lymphoma	4	55/5,206 (10.2)	18/3,469 (6.9)	0.94 (0.49 - 1.78)	6.2 (-78.4 - 50.7)	3157 (387 - NB)
Myeloma	3	20/3,042 (6.3)	10/2,236 (6.0)	1.57 (0.64 - 3.82)	-56.5 (-281.6 - 35.8)	NB (891 - NB)
Myelodysplastic syndromes	2	19/1,405 (13.9)	6/930 (9.0)	0.48 (0.17 - 1.31)	52.3 (-31.0 - 82.6)	276 (174 - NB)
All cancer - medications						
Cytotoxic chemotherapy	4	108/19,380 (5.9)	27/10,035 (3.7)	0.76 (0.47 - 1.24)	23.5 (-24.4 - 53.0)	1433 (636 - NB)
Immunotherapy	4	257/42,833 (5.8)	71/27,466 (3.4)	0.64 (0.48 - 0.87)	35.6 (13.0 - 52.2)	966 (657 - 2634)
Systemic corticosteroids	4	198/27,474 (7.4)	50/16,022 (4.2)	0.61 (0.43 - 0.87)	38.9 (13.1 - 57.0)	698 (476 - 2071)
Solid malignancy – medications						
Cytotoxic chemotherapy	4	93/17,220 (5.8)	26/8,862 (4.0)	0.83 (0.50 - 1.38)	17.0 (-37.9 - 50.0)	2034 (690 - NB)
Immunotherapy	4	213/35,660 (5.8)	59/22,954 (3.4)	0.64 (0.46 - 0.89)	36.2 (11.4 - 54.0)	949 (635 - 3008)
Systemic corticosteroids	4	170/23,840 (7.4)	40/13,846 (3.9)	0.57 (0.39 - 0.84)	42.9 (15.9 - 61.3)	634 (444 - 1716)
Hematologic malignancy – medications & transplants						
Cytotoxic chemotherapy	3	32/3,880 (8.5)	12/2,224 (7.5)	1.33 (0.60 - 2.98)	-33.3 (-197.7 - 40.4)	NB (582 - NB)

© 2025 American Medical Association. All rights reserved, including those for text and data mining, AI training, and similar technologies.

Cancer types*	# sites contributing to pooled analysis	No bivalent vaccination<br><br># events/# people (rate per 1000 person-years)	Bivalent vaccination<br><br># events/# people (rate per 1000 person-years)	Pooled adjusted hazard ratio† (95% CI)	Effectiveness of bivalent vaccination (%) (95% CI)	Number needed to vaccinate (95% CI)
Immunotherapy	4	111/13,284 (8.0)	36/8,801 (5.4)	0.76 (0.49 - 1.17)	24.1 (-17.4 - 50.9)	1037 (490 - NB)
Systemic corticosteroids	4	78/7,272 (10.7)	23/4,618 (6.8)	0.75 (0.44 - 1.27)	25.4 (-27.2 - 56.2)	740 (334 - NB)
Hematologic transplant	2	6/602 (9.5)	2/476 (6.0)	1.01 (0.14 - 7.36)	-1.1 (-636.0 - 86.1)	NB (243 - NB)
Non-immunocompromised						
All	4	1,851/4,838,493 (0.4)	393/1,569,552 (0.3)	0.75 (0.66 - 0.85)	25.5 (15.3 - 34.4)	21137 (15654 - 35106)
<65 years old	4	613/3,482,574 (0.2)	90/844,131 (0.1)	0.82 (0.64 - 1.06)	17.5 (-6.3 - 36.0)	65590 (31947 - NB)
>=65 years old	4	1,238/1,355,919 (0.8)	303/725,421 (0.5)	0.72 (0.62 - 0.83)	28.0 (16.6 - 37.9)	8418 (6226 - 14217)

*See supplemental tables 2 and 3 for definitions. Patients may have more than one diagnosis and/or received more than one type of medication and are included in the analysis of each stratum they qualify for.
†Each site ran a Cox proportional hazards model on their own data with bivalent vaccination as a time-dependent variable and adjusting for age, sex, race and ethnicity, Charlson index, and vaccination status as of the index date (September 1, 2022). Pooled hazard ratios were calculated from the inverse variance-weighted fixed effects of the proportional hazards model results from each site[18].

Other gastrointestinal cancers include esophageal, pancreatic, gall bladder, anus, anal canal, and anorectum cancer

NB = no benefit

© 2025 American Medical Association. All rights reserved, including those for text and data mining, AI training, and similar technologies.

eFigure 1. Effectiveness and number needed to vaccinate to prevent diagnosed COVID-19 in people with cancer and non-immunocompromised persons: One additional dose of monovalent COVID-19 vaccination (booster) compared to primary series only (January 1, 2022 to August 31, 2022) and bivalent COVID-19 vaccine compared to no bivalent vaccine (September 1, 2022 to August 31, 2023), pooled results from four health care systems

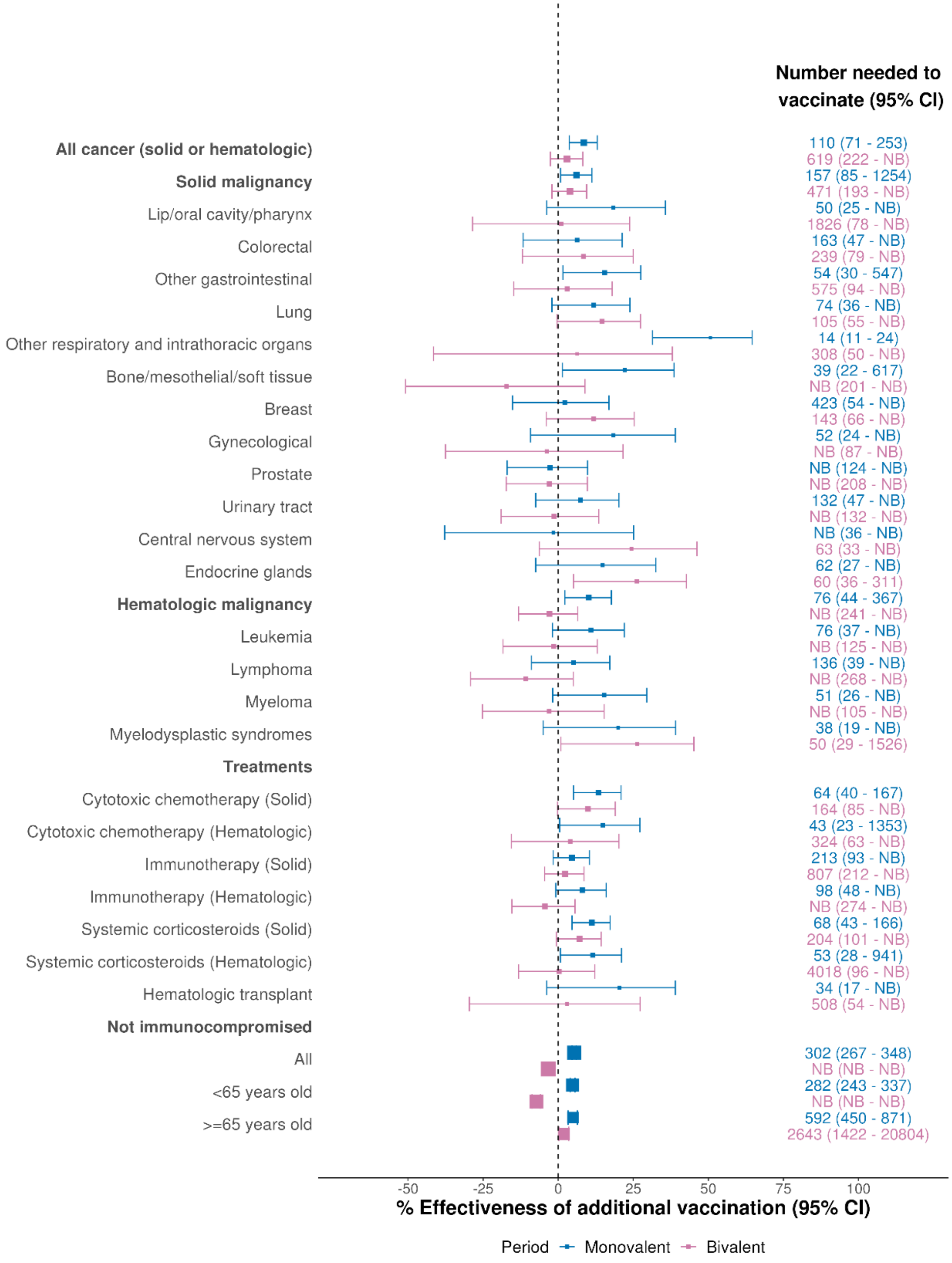

Notes:

1. Pooled vaccine effectiveness = (1 – pooled hazard ratio). Pooled hazard ratios are calculated from the inverse variance-weighted fixed effects of the multivariate proportional hazards model results from each site. Each site ran a Cox proportional hazards model on their own data controlling for age, sex, race and ethnicity, Charlson

© 2025 American Medical Association. All rights reserved, including those for text and data mining, AI training, and similar technologies.

index. For analysis of bivalent vaccinations, the vaccination status as of the index date was also included. See analysis methods for more detail.

2. Patients may have more than one diagnosis and/or received more than one type of treatment in the past year and are included in the analysis of each stratum they qualify for.
3. The size of the square marker is proportional to the size of the population. See Supplemental Tables 4 and 6 for population sizes.

© 2025 American Medical Association. All rights reserved, including those for text and data mining, AI training, and similar technologies.

eFigure 2. Effectiveness and number needed to vaccinate to prevent COVID-19 hospitalization with ICU admission in people with cancer and non-immunocompromised persons: One additional dose of monovalent COVID-19 vaccination (booster) compared to primary series only (January 1, 2022 to August 31, 2022) and bivalent COVID-19 vaccine compared to no bivalent vaccine (September 1, 2022 to August 31, 2023), pooled results from four health care systems

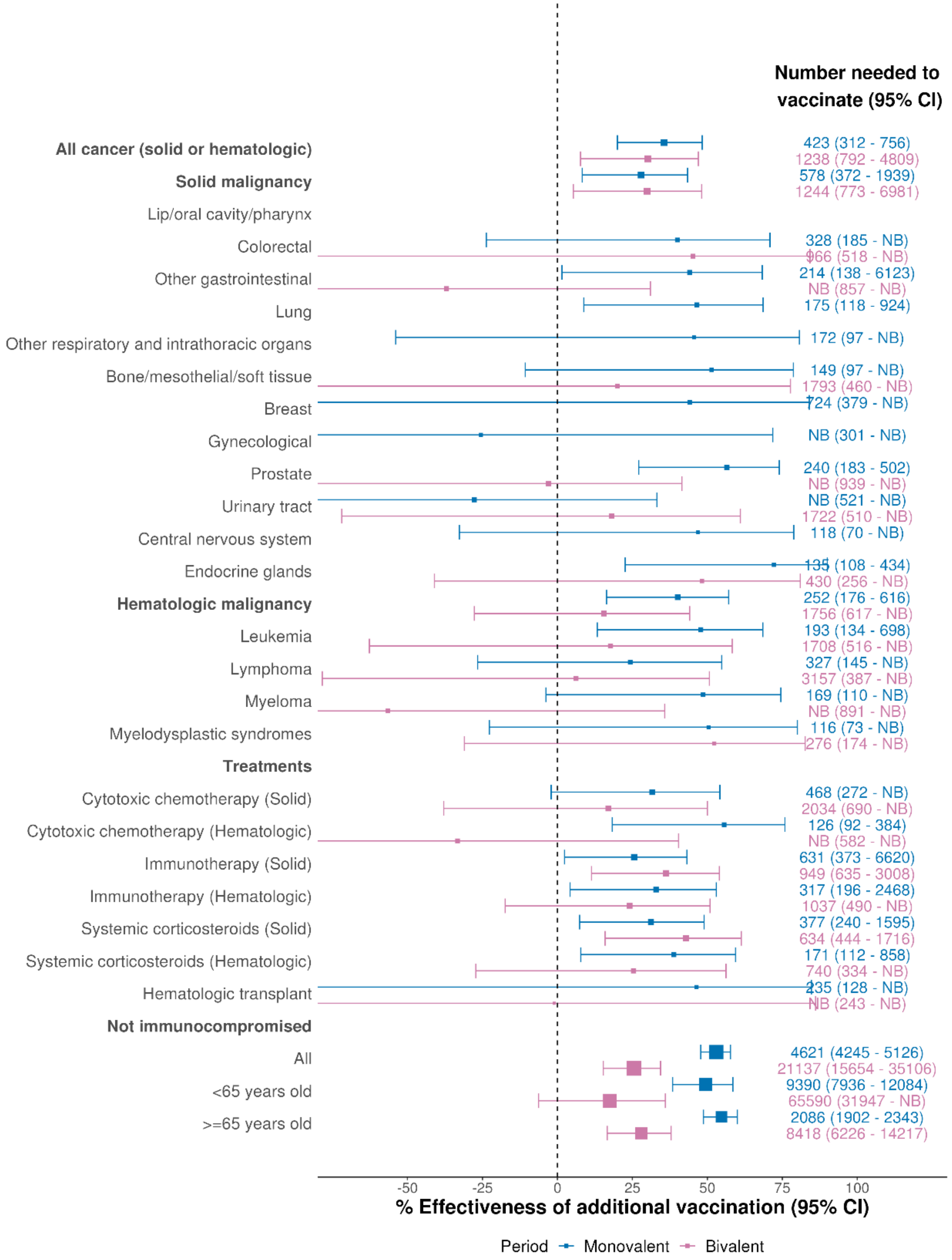

Notes:

1. Pooled vaccine effectiveness = (1 – pooled hazard ratio). Pooled hazard ratios are calculated from the inverse variance-weighted fixed effects of the multivariate proportional hazards model results from each site. Each site ran a Cox proportional hazards model on their own data controlling for age, sex, race and ethnicity, Charlson

© 2025 American Medical Association. All rights reserved, including those for text and data mining, AI training, and similar technologies.

index. For analysis of bivalent vaccinations, the vaccination status as of the index date was also included. See analysis methods for more detail.
2. Patients may have more than one diagnosis and/or received more than one type of treatment in the past year and are included in the analysis of each stratum they qualify for.
3. The size of the square marker is proportional to the size of the population. See Supplemental Tables 5 and 7 for population sizes.

© 2025 American Medical Association. All rights reserved, including those for text and data mining, AI training, and similar technologies.

eFigure 3. COVID-19 vaccine effectiveness in people with cancer for prevention of COVID-19 hospitalization, diagnosed COVID-19 and COVID-19 hospitalization with intensive care unit (ICU) admission: One additional dose of monovalent COVID-19 vaccination (booster) compared to primary series only (January 1, 2022 to August 31, 2022) and bivalent COVID-19 vaccine compared to no bivalent vaccine (September 1, 2022 to August 31, 2023), site-specific results and summary results from four health care systems

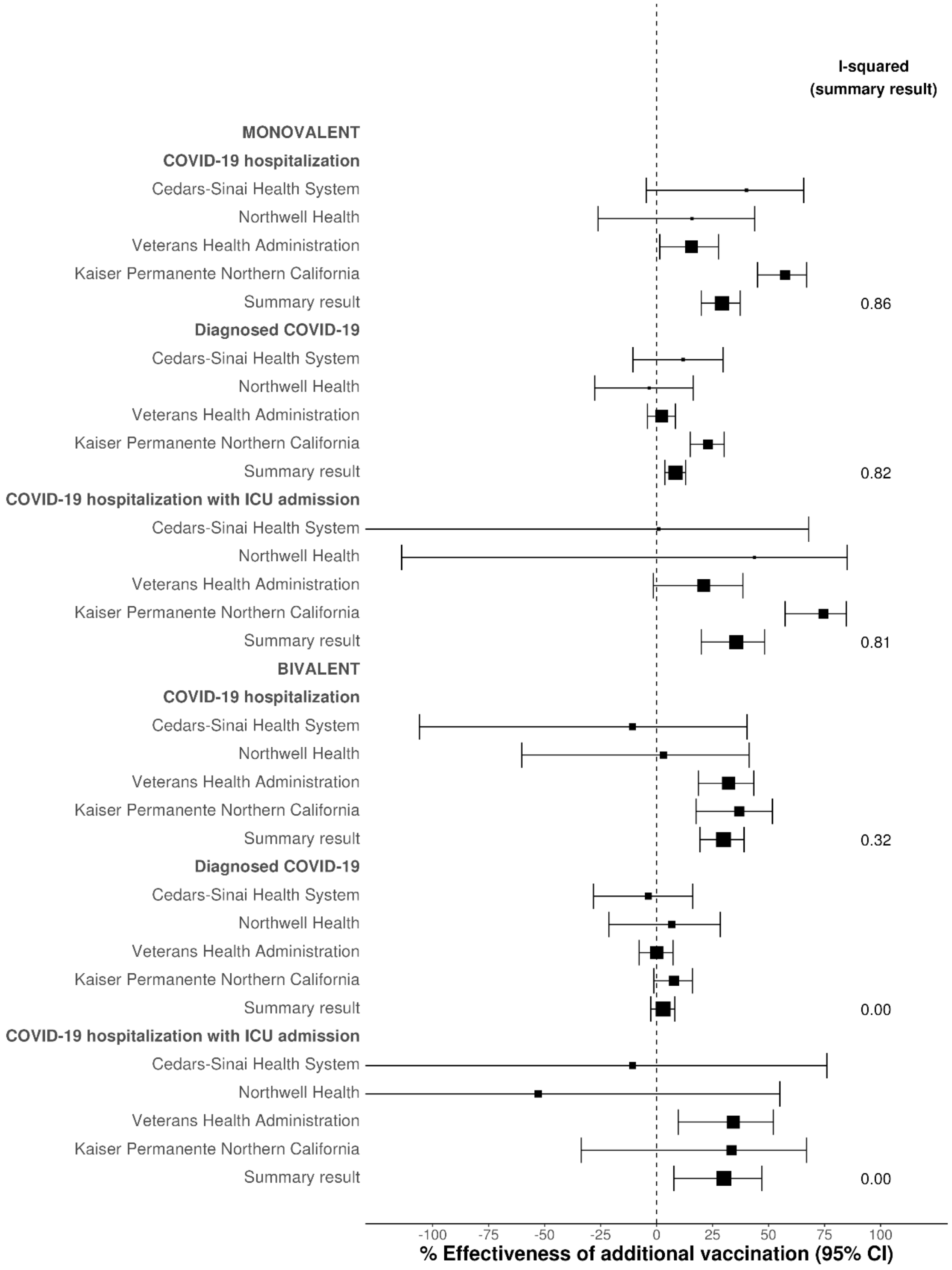

Notes:

1. Pooled vaccine effectiveness = (1 – pooled hazard ratio). Pooled hazard ratios are calculated from the inverse variance-weighted fixed effects of the multivariate proportional hazards model results from each site. Each site ran a Cox proportional hazards model on their own data controlling for age, sex, race and ethnicity, Charlson

© 2025 American Medical Association. All rights reserved, including those for text and data mining, AI training, and similar technologies.

index. For analysis of bivalent vaccinations, the vaccination status as of the index date was also included. See analysis methods for more detail.

2. The size of the square marker is proportional to the size of the population.
3. We present the I^2 statistic, a measure of heterogeneity in meta-analysis, quantifying the proportion of variation in effect sizes across studies due to real differences between studies rather than chance. It's a percentage value, ranging from 0% to 100%, where higher values indicate greater heterogeneity. I^2 is not an absolute measure of heterogeneity and should be interpreted cautiously, especially with small numbers of studies. It can be influenced by factors like the size and weighting of individual studies. https://journals.plos.org/plosone/article?id=10.1371/journal.pone.0039471

© 2025 American Medical Association. All rights reserved, including those for text and data mining, AI training, and similar technologies.
