# Supplementary material for: COVID-19 Vaccine Booster Uptake and Effectiveness Among US Adults With Cancer
Source: JAMA Oncol. 2025 Jul 17;11(9):999–1010. doi: 10.1001/jamaoncol.2025.2020 (PMC12272354; doi:10.1001/jamaoncol.2025.2020)
Supplement: Supplement 2. — Data Sharing Statement [file jamaoncol-e252020-s002.pdf]

Data Sharing Statement

Skarbinski. COVID-19 Vaccine Booster Uptake and Effectiveness Among US Adults With Cancer. *JAMA Oncol*. Published July 17, 2025. doi:10.1001/jamaoncol.2025.2020

Data
Data available: No

Additional Information
Explanation for why data not available: Anonymized data that support the findings of this study may be made available from the investigative team in the following conditions: (1) agreement to collaborate with the study team on all publications, (2) provision of external funding for administrative and investigator time necessary for this collaboration, (3) demonstration that the external investigative team is qualified and has documented evidence of training for human subjects protections, and (4) agreement to abide by the terms outlined in data use agreements between institutions.
